# Views on Antlers Then and Now: Past and Present Concepts of Their Development and Histogenesis

**DOI:** 10.3390/ani16193092

**Published:** 2026-10-01

**Authors:** Uwe Kierdorf, Horst Kierdorf

**Affiliations:** Department of Biology, University of Hildesheim, 31141 Hildesheim, Germany; kierdorf@uni-hildesheim.de

**Keywords:** antler development, cranial appendages, deer, history of antler research, pedicles, regeneration, stem cells, tumors

## Abstract

Antlers are regularly replaced bony appendages that are grown, cast and regenerated from permanent outgrowths (pedicles) of the frontal bones of male deer (and female reindeer/caribou, *Rangifer tarandus*). Their enormous growth rate, periodic epimorphic regeneration, tight association with the reproductive cycle in species from temperate and arctic regions, and low susceptibility to carcinogenesis make antlers highly interesting study subjects for different scientific disciplines, including bone biology, regenerative medicine and cancer research. The exceptional properties of antlers have fascinated scholars since the time of Aristotle, and in the past, many prominent scientists expressed their views on these “improbable appendages”. This contribution reviews some historical concepts related to the development and histogenesis of antlers and contrasts them with our currently held views on these issues. In doing so, we attempt to provide today’s antler scientists with a sense of the long research tradition in the field and to demonstrate how our current ideas on the mechanisms underlying the development and regeneration of antlers have evolved.

## Simple Summary

Antlers are regularly replaced bony structures that develop from permanent outgrowths of the frontal bones (pedicles) of male deer (and female reindeer/caribou, *Rangifer tarandus*). Their enormous growth rate, periodic regeneration, tight association with the reproductive cycle in species from temperate and arctic regions, and cancer resistance make antlers interesting subjects for different fields of biomedicine. Antlers have fascinated scholars for a long time, and the aim of the present paper is to review and compare past and present views on antler development and histogenesis. This can provide today’s antler researchers with a deeper understanding of the research tradition in their field and help them to view their work in an historical context.

## 1. Introduction

*“It seems to me that scientists, in the company of their colleagues in the history and philosophy of science, ought to reexamine with reasonable frequency the map of progress in their chosen fields. In addition to refreshing or updating their own perspectives, another aim is to provide the apprentice with a useful tool for viewing the historical depth, breath, and richness of the issues upon which the field was established”*; Charles E. Dinsmore ([1], p. ix).

Antlers and the permanent frontal outgrowths (pedicles) from which they are grown and cast are a key characteristic and the single most distinguishing feature of the family Cervidae [2,3,4]. The many exceptional properties of antlers prompted Richard J. Goss (1925–1996), one of the most prominent antler researchers of the 20th century, to characterize them as “improbable appendages” and to state that “*if they had not evolved in the first place they would never have been conceived even in the wildest fantasies of the most imaginative biologists*” ([2], p. 1). Regarding the extraordinary features of antlers, in his *Handbuch der Vergleichenden Anatomie* Johann Friedrich Blumenbach (1752–1840) had almost 180 years earlier stated that the “*annual regrowth of antlers is, for several reasons, one of the strangest phenomena of animal physiology. It is one of the most conspicuous examples of a) the power of nutrition and the resulting rapid growth in warm-blooded animals […] b) the partial lifespan of the part of an animal that is totally independent of the age of the entire animal […] c) the variability in the caliber of individual blood vessels […] and d) the curious so-called consensus that exists between antler growth and reproductive activity*.” ([5], pp. 34–35; authors’ translation). Here, Blumenbach highlights several aspects of antlers that are currently still in the focus of research. Over the last centuries, many scientists developed a strong interest in the cranial appendages of deer; however, as stated by Goss [2], the number of scholars seriously involved in antler research at any one time has always been low. More recently, however, the translational aspects of antler research have gained increasing interest from the research community, as findings on antlers may hold important clues for improving wound healing and regeneration [6,7,8], cancer treatment [9,10,11,12], and the therapy of osteoporosis [13] in humans.

The aims of the present paper are (1) to give a brief overview on the development and structure of the particular type of “headgear” [14] present in deer, (2) to discuss some important aspects of pedicle and antler development that make them interesting objects of scientific inquiry, and (3) to provide a historical review of the changing views on the growth and regeneration of antlers. Following the reasoning cited in the motto of this paper, the main goal of our approach is to provide today’s antler scientists with a sense of the long research tradition in their field of study and make them aware that many questions regarding the cranial appendages of deer that are discussed today have already been turned over in the minds of their predecessors. We do not attempt to provide a complete history of antler research, but restrict ourselves to a discussion of some basic aspects of antlers that have been a focus of our own work in recent decades.

## 2. Methods

A literature search on antler-related topics was performed using the bibliographic databases Web of Science (Clarivate^TM^, period 1945–2026) and PubMed (NIH, period 1923–2026). The search strategy included combinations of “antler” with one of the following search terms (in alphabetical order): “abnormal*”, “androgens”, “appendage”, “blastema”, “bone”, “blood supply”, “cancer”, “cartilage”, “casting”, “castrates”, “cycle”, “development”, “differentiation”, “evolution”, “female*”, “growth”, “histogenesis”, “histology”, “history”, “hormones”, “mineralization”, “nerves”, “neural crest”, ossification”, “osteoporosis”, “pedicle”, “periosteum”, “photoperiod”, “proteomics”, “regeneration”, “regulation”, “reproduction”, “stem cells”, “structure”, “transcriptomics”, “translational”, “transplantation”, “tumor”, “velvet”, and “wound healing”. The retrieved results were assessed regarding their relevance for the present study, and the publications deemed relevant were included.

Given the historical nature of the topic, we placed a focus of our study on the analysis of earlier (pre- and early to mid-20th century) works. Most of these publications could not, however, be retrieved from the two mentioned databases, as was also the case with much of the older non-English literature. Instead, the inclusion of these publications in the study was possible due to their accessibility via Google Scholar and/or their presence in the authors’ literature archive.

## 3. Overview of the Cranial Appendages of Deer

Four of the six living families of ruminants possess cranial appendages, which are known as horns in the Bovidae, pronghorns in the Antilocapridae, ossicones in the Giraffidae, and pedicles plus antlers in the Cervidae. Cranial appendages were also present in the extinct ruminant families Climacoceratidae and Palaeomerycidae, while they are missing in the Moschidae and Tragulidae [14,15,16]. There exists a longstanding controversy about whether the various types of cranial appendages in ruminants evolved separately in the different lineages or have a single evolutionary origin [14,15,17]. More recent genetic studies suggest a common origin of cranial appendages from neural crest stem cells in the infraorder Pecora (Ruminantia except Tragulidae), followed by independent losses of these appendages in the Moschidae and in the antlerless water deer (*Hydropotes inermis*) within the Cervidae [16,18].

**Figure 1:**
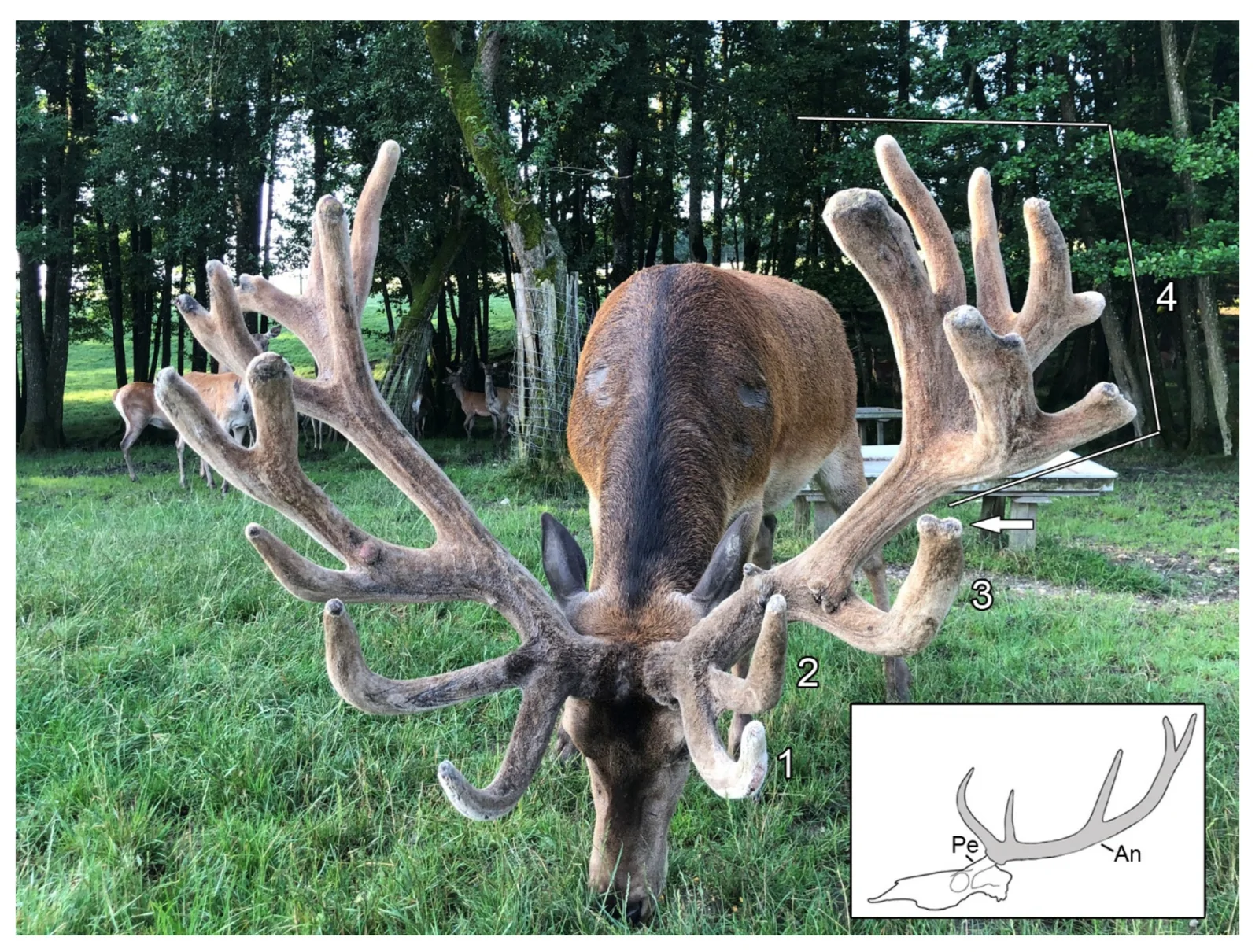
Red deer (*Cervus elaphus*) stag at the end of the antler growth phase. The antlers are still covered by velvet. The stag was kept in an enclosure under highly favorable nutritional conditions and produced profusely branched antlers (7th antler set or “head”) of 16.8 kg dry weight. On the left antler, the brow tine (1), bez tine (2), trez tine (3) and the crown with multiple tines (4) are identified. For antler tine terminology, see [25]. Note double branching of the right trez tine and tendency for dichotomous splitting of some tine tips (arrow), denoting a potential for further antler branching. The inset image illustrates the topographic relationship and difference in length between pedicle (Pe) and antler (An, with five tines) in a red deer stag.

**Figure 2:**
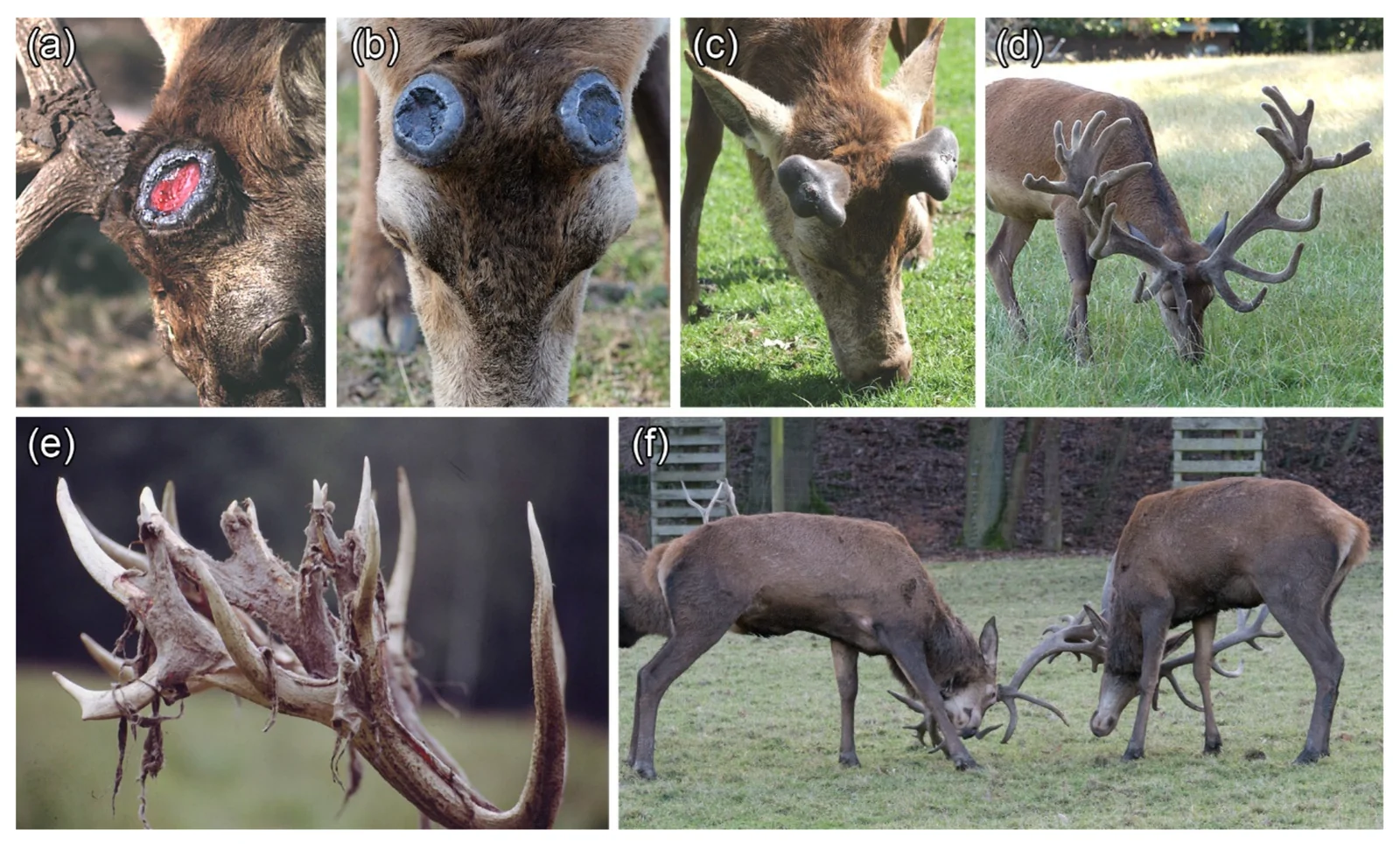
Different stages of the antler cycle in red deer (*Cervus elaphus*). (**a**) Stag immediately after casting of the left antler, with a fresh bone wound on the top of the left pedicle. Note rim of swollen skin around the wound surface. The right antler is still attached to the pedicle. (**b**) Shortly after antler casting, the wound surfaces on the top of the pedicles are covered with scabs. (**c**) Early stage of antler regeneration with presence of separate growth centers for the main beams and brow tines. Note persisting scabs in the furcation area between main beam and brow tine. (**d**) Fully regrown antlers shortly before velvet shedding. (**e**) Velvet shedding exposes the bare bony (hard) antlers. The stripped velvet hangs down in tatters and is subsequently lost. (**f**) Two stags using their hard antlers in a sparring contest.

**Figure 3:**
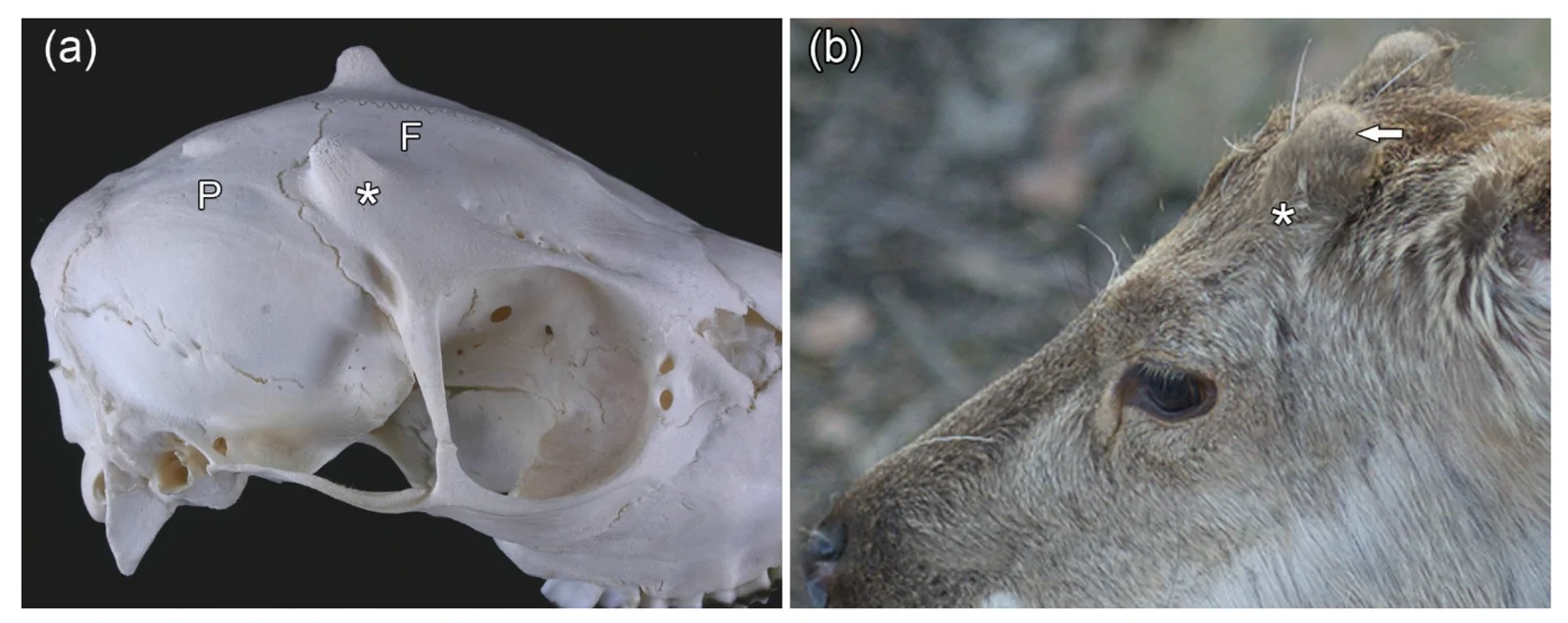
Primary cranial appendage formation in deer. (**a**) Cranium of a male European roe deer (*Capreolus capreolus*) fawn, aged about 4 months, showing incipient pedicles (asterisk) developing as frontal apophyses. F—frontal bone, P—parietal bone. (**b**) Male sika deer (*Cervus nippon*) fawn, aged about 9 months, with pedicles (asterisk) covered by normal scalp skin and incipient first antlers (arrow) covered by velvet.

**Figure 4:**
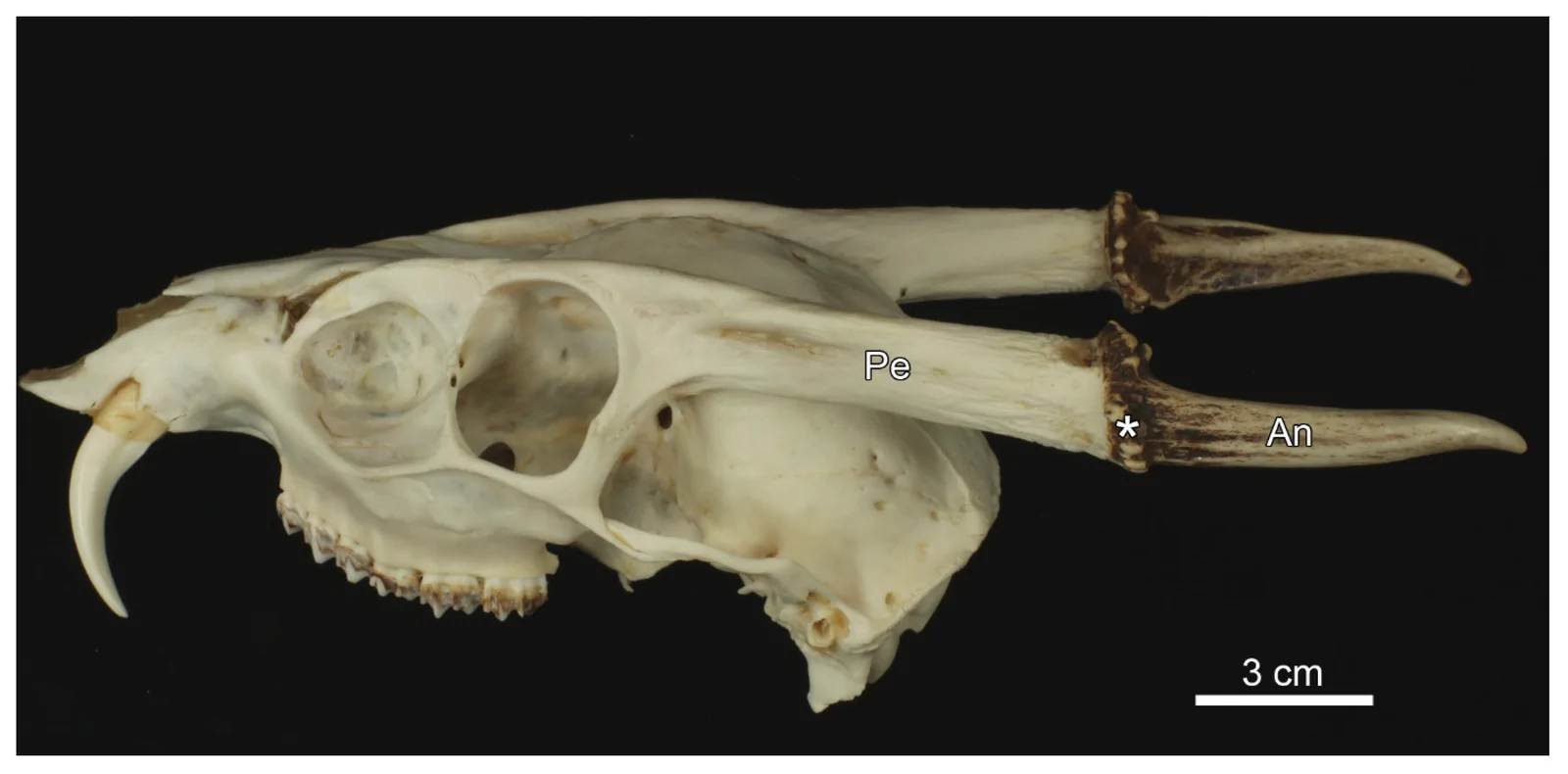
Cranium of a male Reeves’ muntjac (*Muntiacus reevesi*) with pedicles (Pe) that exceed the antlers (An) in length. The presence of burrs or coronets (asterisk) at the base of the antlers indicates that they are regenerated ones.

The bony cranial appendages of deer differ from those of other ruminants in that they consist of permanent basal portions, the pedicles, and regularly replaced distal portions, the antlers (Figure 1 and Figure 2). Pedicles develop postnatally as paired outgrowths (apophyses) of the frontal bones (Figure 3a) and are covered by normal scalp skin (Figure 3b) [2,19]. In most extant deer species, the pedicles are much shorter than the antlers (Figure 1, inset), but this is not without exceptions. Thus, in muntjacs (*Muntiacus*) the pedicles are very long and can exceed the antlers in length (Figure 4). Long pedicles and short antlers are also a characteristic of several Miocene cervids, such as *Procervulus*, *Euprox* and *Lagomeryx* [20].

The antlers form on top of the pedicles, and during their growth period of a few months (typically 3.5 to 5, depending on the species) are covered by a special type of skin, referred to as velvet (Figure 1, Figure 2c,d and Figure 3b). Velvet differs from normal scalp skin in several aspects, including a thickened epidermis, the lack of arrector pili muscles and sweat glands, and the presence of large, multi-lobed sebaceous glands [21,22,23]. There can be considerable annual variation in the length of the velvet antler period in single individuals, as has for instance been documented in Reeves’ muntjac (*Muntiacus reevesi*) [24].

Except for the reindeer/caribou (*Rangifer tarandus*), only male deer regularly grow pedicles and antlers, which therefore constitute male secondary sexual characters [2,4,26]. The antler cycle of male deer from temperate and arctic regions is linked to their reproductive cycle [27,28,29,30], and their reproductive and (dependent) antler cycles are tightly controlled by the photoperiod [31,32,33,34]. The first experiments demonstrating the effects of day length on the antler cycle were performed on red deer (*Cervus elaphus*) by Zbigniew Jaczewski (1928–2006) in Poland in the early 1950s [35]. Later studies on sika deer (*Cervus nippon*) [31] and fallow deer (*Dama dama*) [34] showed that males exposed to a threefold acceleration of the annual light cycle undergo three antler cycles in a calendar year. However, the antlers produced under these conditions were stunted compared to those produced under normal photoperiodic conditions. Repeated formation of two antler sets per year has been observed in a free-ranging red deer stag exposed to normal photoperiodic conditions [36]. Also in this case, the extra antlers were stunted compared to those grown previously in the course of regular antler cycles. Premature antler casting and extra-seasonal antler growth were attributed to an abnormal shift in the seasonal fluctuation of testosterone production in the stag.

Contrary to cervids inhabiting higher latitudes, the antler cycle of different deer species native to tropical and subtropical regions, with only small changes in daylength throughout the year, has been reported to be aseasonal and asynchronous [2,37,38]. Thus, males in different stages of the antler cycle can be simultaneously present in a population. Moreover, males of tropical/subtropical deer species are fertile all year round and their reproductive and antler cycles are uncoupled [2,39,40,41,42]. This means that they can maintain spermatogenesis and successfully breed regardless of the condition of their antlers. In the case of Reeves’ muntjac, it has been suggested that this uncoupling may be due to a difference in the threshold levels of testosterone required for spermatogenesis and for antler maturation and velvet shedding [40]. Another special feature of the antler cycle reported for some tropical deer is that they may retain their hard antlers for extended periods of time [2].

The periodic regrowth of antlers throughout a deer’s life is a unique case of repeated regeneration of a large bony appendage in a mammal, a group with otherwise very limited capacity for appendage regeneration [43,44,45,46,47]. The only other known instance of epimorphic appendage regeneration in postnatal mammals is digit tip regeneration [48,49]. In mice, amputation through the distal half of the terminal phalanx, which is associated with the nail organ, triggers regeneration, while amputation at further proximal levels does not [48].

The growth of the first antlers, typically unbranched “spikes”, constitutes a continuation of pedicle growth and starts once the latter have attained a species-specific threshold length [2,19,50,51]. Formation of the so-called primary cranial appendages of deer therefore involves the transition from a permanent (pedicle) to a deciduous (antler) bony structure (Figure 3b). Contrary to antlers grown during later life, the development of the first set of antlers is thus not a regenerative event, and it is with the casting of its first antlers that a deer enters into the annual cycle of antler replacement. The antlers formed during later life tend to increase in size and tine number until prime age (Figure 1). The branching pattern of antlers in different deer species has been repeatedly studied in an attempt to identify homologous elements and thereby to analyze their potential for reconstructing cervid systematics [25,52,53,54].

In the past, it has been hypothesized that in the course of cervid evolution, deciduous antlers were preceded by permanent antler-like structures (e.g., [55]) and that the different antler characteristics were gradually acquired during evolution (for a review of these views, see [20]). However, recent studies provided convincing evidence that right from their first appearance in the fossil record, antlers were deciduous appendages, and that all features characterizing today’s antlers were already present in the earliest antlers from the Miocene [20,56]. Antlers have therefore been characterized as a synapomorphic trait complex of the family Cervidae [25].

## 4. The Link Between the Reproductive Physiology of Deer and Their Cranial Appendages

One of the most conspicuous aspects of antlers is certainly their tight association with the reproductive cycle in deer species inhabiting higher latitudes, a condition referred to as “consensus” by Blumenbach [5]. This term was also used by Arnold Adolf Berthold (1803–1861) in his treatise on the growth, casting and regeneration of antlers [50]. Berthold is one of the founders of experimental endocrinology, who performed the classic experiment of transplanting testes into the abdominal cavity of castrated male chickens, thereby preventing the occurrence of castration effects [57]. The outcome of this experiment led him to conclude that the consensus between testes and body condition “*is caused by the productive relationship of the testes, that is, their influence on the blood and further the influence of the blood on the general organism*” ([57], p. 46; authors’ translation).

Scientific interest in the relationship between the reproductive organs and antlers of male deer dates back to the 4th century BCE, when Aristotle (384–322 BCE) described the outcome of castration on the antlers, depending on the age of the animal at which the testicles are removed. In his *History of Animals*, he states that if “*deer are castrated when they have as yet no horns because of their age, they cease to grow horns; but if they already have them when castrated, the size of the horns remains the same and they do not cast them*” ([58], p. 401). As will be detailed below, the second half of this statement is correct only if it refers to males castrated during the velvet antler phase.

Pedicle growth is triggered postnatally by a temporary increase in circulating testosterone in young male deer [28,59,60]. This temporary testosterone surge activates a specialized periosteum, known as antlerogenic periosteum (AP) [2], which overlies the prospective pedicle site (see below). Castration of pre-pubertal young males prevents this rise in circulating testosterone and therefore permanently precludes pedicle and subsequent antler growth [2,27,33]. This outcome of early castration of young male deer was already correctly described by Aristotle [58].

The above description does, however, not hold for *Rangifer tarandus*, the only species in which antlers are grown by both sexes. In this species, early postnatal (day 7 after birth) pedicle initiation was observed in both male and female calves, and velvet transformation of the skin occurred at the age of 4 to 6 to six weeks [61]. A further study demonstrated that early postnatal gonadectomy in male and female individuals that had not yet produced palpable pedicles did not preclude pedicle and antler development [62]. This indicates that the formation of primary cranial appendages in reindeer/caribou does apparently not depend on stimulation by gonadal hormones. In *Rangifer tarandus*, antlers are annually cast and regrown by both intact and castrated males and by females [2,62]. As in other deer species, the annual antler cycle of intact male reindeer/caribou is controlled by seasonal fluctuations in blood testosterone levels [29,61,63], and castration of males in hard antler causes premature antler casting [29]. In contrast, the annual antler cycle of female reindeer/caribou is regulated by estradiol, which controls antler bone mineralization, velvet shedding and the suppression of casting of the hard antlers [64]. In castrated males of the species, the antler cycle is thought to be controlled by adrenal steroids [65].

In red deer, Lincoln [66] reported the appearance of “pedicles” already in male fetuses. However, it was later shown that the conspicuous swellings present on the fetal heads at the future pedicle sites represent local thickenings of the epidermis that regress during later stages of gestation [67]. Importantly, neither bony outgrowths nor a local thickening of the periosteum (future AP) underneath the epidermal swellings on the heads of the fetal deer were observed [67]. Li and Suttie [67] compared these epidermal thickenings to the apical ectodermal ridge, a strip of ectodermal cells that runs along the distal margin of the vertebrate limb bud and develops into a major signaling center during limb development [68,69]. It therefore seems inappropriate to use the term pedicles for these local epidermal swellings that are considered to be formed in response to a testosterone surge during a period of increased testicular activity in the fetus [66]. However, formation of these swellings may well be an initial step in the developmental cascade eventually leading to onset of pedicle formation in juvenile male deer.

According to a detailed histological study by Georg B. Gruber (1884–1977) in the European roe deer (*Capreolus capreolus*), the first sign of postnatal pedicle formation in fawns is a resorption on the inner aspect of the outer table (lamina externa) of the frontal bone from the marrow spaces of the diploë at the future sites of pedicle growth [70]. When this resorptive process has been ongoing for some time, periosteal (AP) bone apposition onto the outer aspect of the frontals starts. According to our knowledge, corresponding findings have thus far not been reported from other deer species.

During antler growth, testosterone levels are low [27,29,30,71]. It has been stated that during the first weeks of antler growth, a red deer stag may therefore be regarded as “*almost a functional castrate*” ([71], p. 932). This statement notwithstanding, the precise role of androgens in velvet antler growth has been a matter of controversy. Thus, it has been suggested that small amounts of circulating androgens are needed to stimulate antler growths and/or that the onset of antler regeneration is triggered by a brief testosterone pulse [27,72,73]. In contrast, based on the results of antiandrogen (cyproterone acetate) treatment of intact male deer, other authors concluded that antler growth does not require stimulation by androgens [30,74,75]. However, when castrated fallow bucks were additionally treated with cyproterone acetate, their antler growth was significantly reduced and their plasma testosterone concentration significantly lowered compared to castrates not receiving the antiandrogen [76]. Based on these findings, it was suggested that androgen values above a certain low threshold level may be a prerequisite for antler growth at a normal rate. Regarding the mechanism(s) of antler growth promotion, there is strong in vivo and in vitro evidence that the rapid proliferation of the mesenchymal cells in the tip regions of growing antlers is mediated by insulin-like growth factors (IGFs), with IGF-1 being the main antler growth stimulating hormone [77,78,79,80,81].

The processes involved in the control of antler morphogenesis are still poorly understood. Based on observations that unilateral injury to growing antlers may also affect the morphology of ipsilateral antlers produced in subsequent years, a phenomenon they referred to as “trophic memory”, Anthony B. Bubenik (1913–1995) and his son George A. Bubenik (1942–2018) postulated the existence of a separate “trophic nerve center” or “antler growth center” for each antler that controls morphogenesis [82,83,84,85]. These hypothetical bilateral control centers, which must not be confused with the antler growth centers present at the tips of regenerating antlers [86], were supposed to be located in the hypothalamus and to exert their influence on the growing antlers via neuronal or neurohumoral pathways [83,84]. The Bubenik hypothesis of the control of antler morphogenesis by trophic centers in the brain has not been widely accepted, and the focus of research has since switched to local mechanisms of antler growth control [45]. Some of these studies have suggested a role for retinoic acid, a vitamin A derivative, in the differentiation of antler stem cells and the local regulation of antler growth and morphogenesis, and [87,88,89,90].

A steep rise in blood testosterone concentration at the end of the antler growth period causes full mineralization of the antler bone and elicits the so-called fraying behavior that leads to velvet shedding, i.e., the removal of the velvet from the antler [2,27,28,29,59]. Antler maturation and fraying behavior are independently triggered by testosterone, which in special cases may lead to velvet shedding from immature antlers [27,91,92]. “Fraying behavior” has even been described in a European roe buck that lacked antlers as a result of bilateral surgical removal of the AP [93].

Velvet shedding exposes the bare bony antlers, which are referred to as hard antlers and used, among other things, for sparring/fighting between males (Figure 2f) [29,94]. Traditionally, hard antlers have been considered dead bone [2,50,95,96]. This view was, however, challenged by some authors, who argued that antlers survive velvet shedding and are supplied with blood through vascular connections with the pedicles [97,98,99]. Furthermore, these authors suggested ongoing bone formation and remodeling in hard antlers. The notion of a functional blood supply of hard antlers has been accepted by some other authors [100].

**Figure 5:**
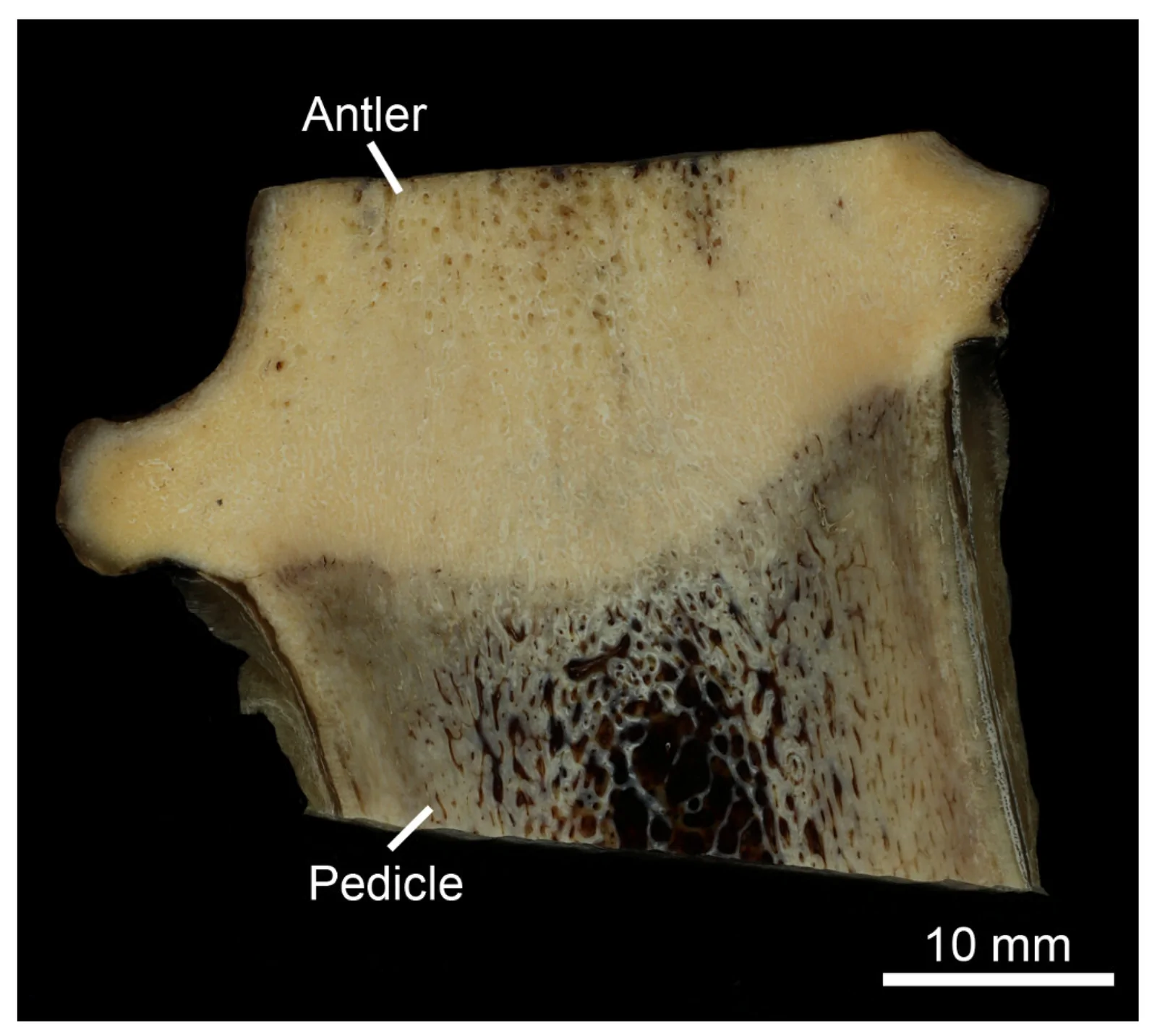
Longitudinal section of the distinct pedicle-hard antler boundary region in a 2yr-old red deer (*Cervus elaphus*) stag about 3 months after velvet shedding. Note the dense ossification of the antler base and the lack of vascular connections between pedicle and hard antler. Also note that the periosteum is attached to the pedicle, while the hard antler is a bare bone.

More recent studies, however, clearly demonstrated that hard antlers are dead structures that die off following the cessation of blood supply at velvet shedding, thereby corroborating the traditional view. Thus, it was shown (1) that antlers rapidly dry out after velvet shedding [101], (2) that the residual fluid initially found in antlers after velvet shedding contains no functional blood cells, (3) that hard antlers become invaded by bacteria, (4) that no viable osteocytes are present in hard antlers [102], and (5) that, contrary to injections during the velvet antler stage, intramuscular injection of oxytetracycline in red deer stags after velvet shedding does not produce fluorescent labels in antler bone [103]. According to George B. Wislocki (1892–1956), first signs of necrosis in the antler’s marrow spaces can already be observed in the late velvet stage as a consequence of impaired circulation [96]. The dense ossification of the antler base and the lack of vascular connections between pedicle and hard antler is illustrated in Figure 5.

**Figure 6:**
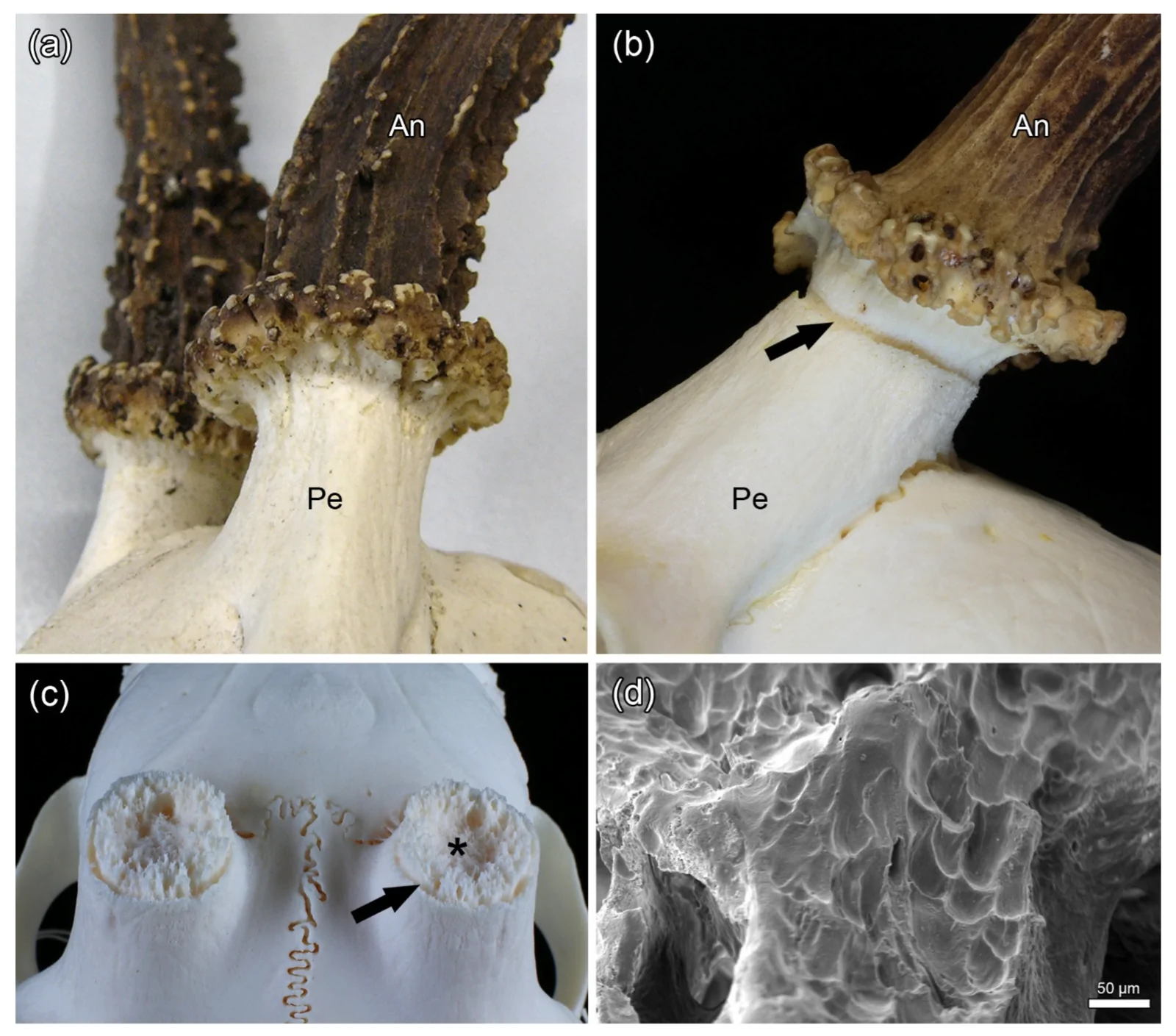
Morphological findings in the pedicles of the European roe (*Capreolus capreolus*) related to the process of antler casting. Antler casting in adult roe bucks typically takes place in October/November. An—antler, Pe—pedicle. (**a**) In a buck that died in late spring (15 June), the pedicle surface shows no signs of bone resorption and the antlers are firmly attached to the pedicles. (**b**) In a buck that died in autumn, a ring-like furrow (arrow) caused by bone resorption is visible in the distal pedicle. (**c**) Fresh casting surfaces on the top of the pedicles. Note extended resorption spaces (asterisk) in the pedicle interior, ring-like furrow along the pedicle surface (arrow) and numerous bony spicules on the fracture plane. (**d**) Scanning electron micrograph (secondary electron mode) of a portion of the fracture plane at the top of the pedicle, showing that it is densely covered with Howship’s lacunae.

The hard antlers remain firmly attached to the living pedicles as long as blood testosterone concentrations are high (Figure 6a) [27,29,30,104]. A pronounced drop in testosterone after the rut initiates the onset of intense osteoclastic activity in the distal pedicle (Figure 6b,c), which eventually causes the antlers to fall-off, a process known as antler casting [70,105,106,107]. In his *Lectures on Comparative Anatomy*, Everard Home (1756–1832) characterized antler casting as “*perhaps the most beautiful instance in nature, of a bone being formed for a temporary use, and cast off by absorption as soon as the purpose is answered for which it was intended*.” ([95], p. 67).

The resorptive process in the pedicle was described in detail by Albert Kölliker (1817–1905) in his seminal work on bone resorption [105], in which he also introduced the term osteoclast (in the treatise still written “*ostoklast*”) into the scientific literature. The bone resorption leading to antler casting takes place both in the interior of the pedicle and along its periphery. The latter process leads to the formation of a ring-like furrow (Figure 6b) that later unites with the resorption spaces in the pedicle’s interior. Osteoclastic activity in the pedicle interior causes an expansion of the Haversian canals in a more or less horizontal plane and the formation of extended resorption spaces [105,106,107,108]. These resorption spaces, termed resorption sinuses by Kölliker [105], eventually become confluent (Figure 6c). The intense osteoclasia in the distal pedicle results in a progressive weakening of the connection between antler and pedicle, and finally the antler, along with a small distal pedicle portion, breaks off from the pedicle due to its own weight or following a minor mechanical impact. The fracture planes of both the cast antler and the pedicle are densely covered with Howship’s lacunae (Figure 6d) [105,106,107,109]. Shortly after casting, the resorption lacunae within the pedicle still contain osteoclasts, whose ongoing activity causes a smoothing of the pedicle’s fracture plane [105,106,110].

There is evidence that the border between living (pedicle) and dead bone (hard antler) is instable. Thus, it has been observed that when hard antler casting in red deer stags was inhibited by treatment with exogenous testosterone or estradiol, the border between dead and living bone gradually shifted proximally within the pedicle, a phenomenon referred to as “die-back” [29].

Antler casting has been characterized as a kind of autotomy [2]. However, when compared to the self-induced loss of the lizard tail, the best-known example of autotomy in vertebrates [111,112], certain differences between this process and antler casting are apparent [113]. First, in the case of caudal autotomy in lizards, a living body part is detached, while the cast antler is a dead structure. Second, contrary to antler casting, caudal autotomy is not brought about by osteoclastic activity. Third, caudal autotomy occurs along a pre-determined separation plane, while antler casting takes place at a level directly proximal to the border between living and dead bone, the location of which can vary and in exceptional cases can be located in the antler [113]. Thus, it is considered more appropriate to characterize antler casting as an example of sequestration of dead bone, as it occurs under certain pathological conditions [113].

**Figure 7:**
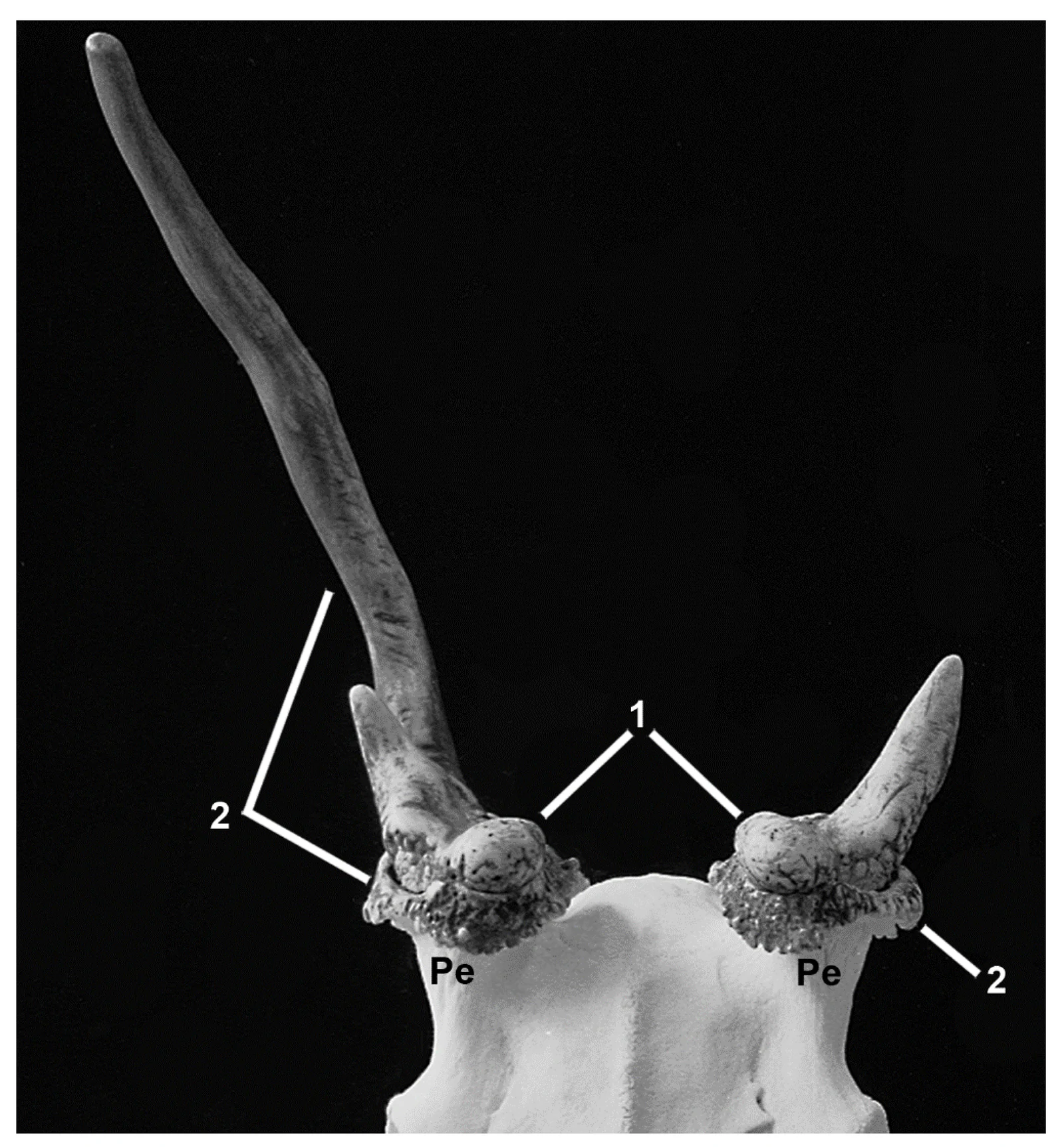
Bilateral double-head antler formation in a fallow buck (*Dama dama*), anterior view of the cranium. The first antlers (1) were not cast, and renewed antler growth caused the formation of a second antler set (2) from the pedicles (Pe). While on the left side, the second antler is limited to a burr-like rim proximal to the first antler, on the right side a beam developed from the basal portion of the regenerated antler. Note presence of a narrow furrow between the two successively formed antlers on each side. These furrows formed when the velvet was shed from the regenerated antlers.

The drop in circulating testosterone that initiates the process of bone resorption leading to antler casting also triggers the onset of antler regeneration, which proceeds irrespective of whether or not the old antlers are cast. When the previous antlers are not (or belatedly) cast, renewed antler growth leads to the presence of two successively produced antlers emanating from the same pedicle, a phenomenon known as double-head antler formation (Figure 7) [114,115,116,117]. In the hunting literature, even the occurrence of three antlers grown during successive cycles from a pedicle has been reported [118]. The growth of a new antler despite the presence of its predecessor proves that antler regeneration is not caused by the casting of the previous (hard) antler. Thus, it is not the casting wound on the surface of the pedicle that triggers antler regeneration, as was suggested by some authors, e.g., [119]. Rather, antler regeneration is considered to be triggered already by the death of the antlers following the cessation of blood supply at velvet shedding, which precedes antler casting by several months [115,116]. An immediate onset of antler regeneration following velvet shedding and subsequent antler death is, however, suppressed by high circulating testosterone levels. Goss [2,43,120] repeatedly emphasized that, like in other cases of epimorphic regeneration, the presence of a healing skin wound is indispensable for the initiation of antler regeneration. Antler regrowth differs from other examples of epimorphic appendage regeneration in vertebrates in that the creation of the skin wound (at velvet shedding) occurs several months prior to that of a bone wound (at antler casting) and that the process of skin wound healing is therefore postponed for a considerable period of time [47].

Castration of male deer carrying velvet antlers prohibits full antler maturation and velvet shedding. In consequence, the antlers remain permanently viable and, as was correctly stated by Aristotle [58], are not cast. In individuals carrying hard antlers, the drop in circulating androgens following castration causes premature antler casting and the formation of a new set of permanently velvet-covered antlers that are kept throughout life [121,122,123,124].

It has been reported that antler growth in castrated European roe deer males is not permanent but shows a certain periodicity [121]. Moreover, the antlers of castrated male deer react to sex steroid administration like normal velvet antlers. Thus, treatment with androgens or estrogens (at a sufficient dose) of castrated males carrying velvet antlers causes growth cessation and full mineralization of antler bone, followed by velvet shedding. When the sex steroid levels in blood drop below a threshold, these antlers are cast and a new set of permanently velvet-covered antlers is produced [121,122,123].

**Figure 8:**
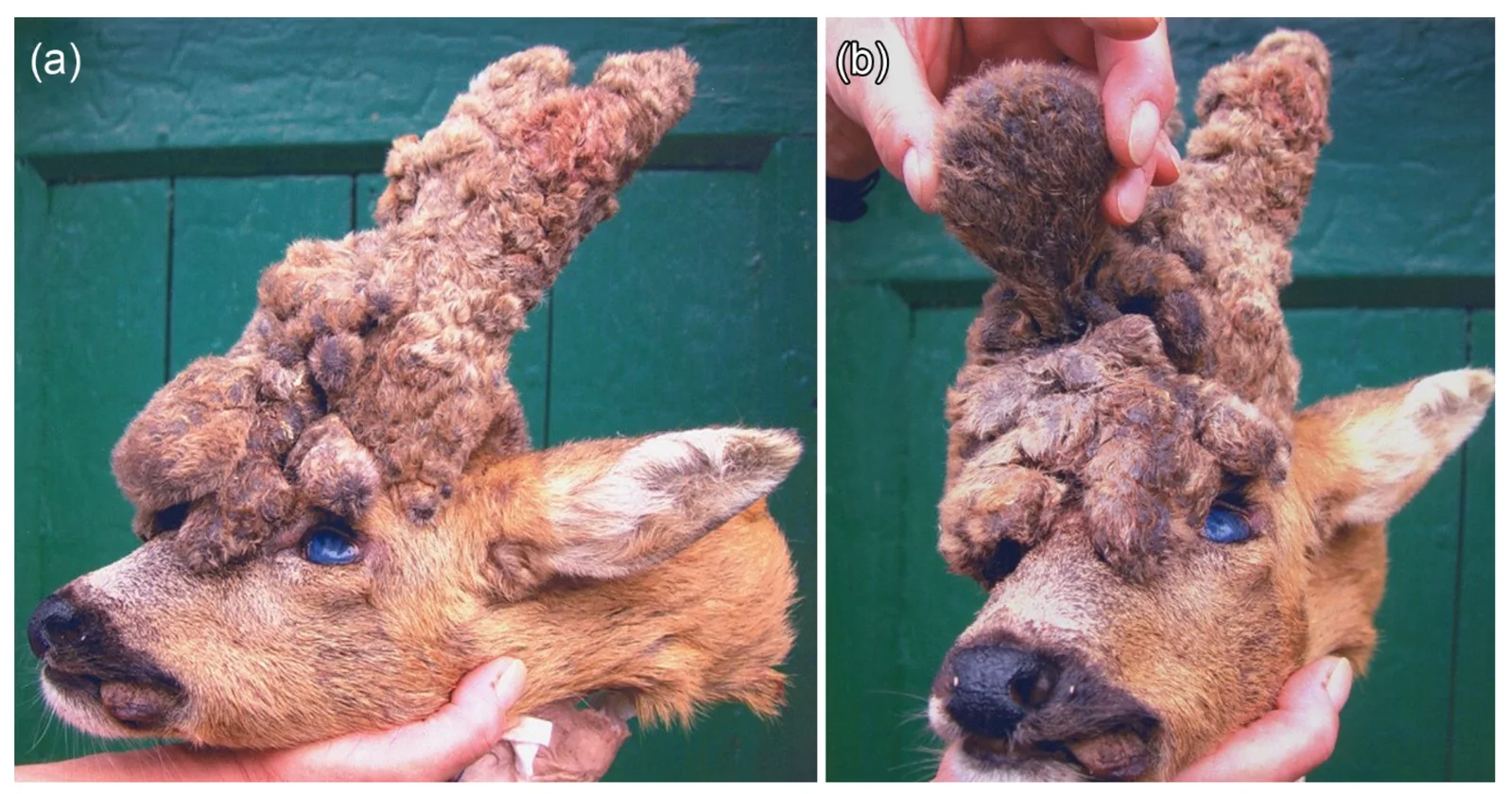
Peruke antlers of a European roe buck (*Capreolus capreolus*). (**a**) Lateral view of the head. (**b**) Anterior view of the head showing pendulous skin protuberances of the peruke antlers.

A conspicuous feature of the antlers of castrated or hypogonadal male deer is the formation of abnormal outgrowths [65,84,118,121,122,125,126,127,128,129,130,131,132,133,134,135,136,137]. The intensity of the antler overgrowth caused by the lack of androgens shows considerable interspecific variation, and in general tends to be higher in members of the subfamily Capreolinae than in those of the subfamily Cervinae. The abnormal growth is particularly prominent in the European roe deer, where castrated or hypogonadal males produce bizarre structures that are referred to as peruke antlers (Figure 8) because of their resemblance to a wig [73,109,117,118,119]. Peruke-like castrate antlers are sometimes also observed in other deer species, e.g., red deer [65]. In deer of the genus *Odocoileus*, individuals with abnormal antlers caused by testosterone insufficiency, are known as cactus bucks [2,136].

**Figure 9:**
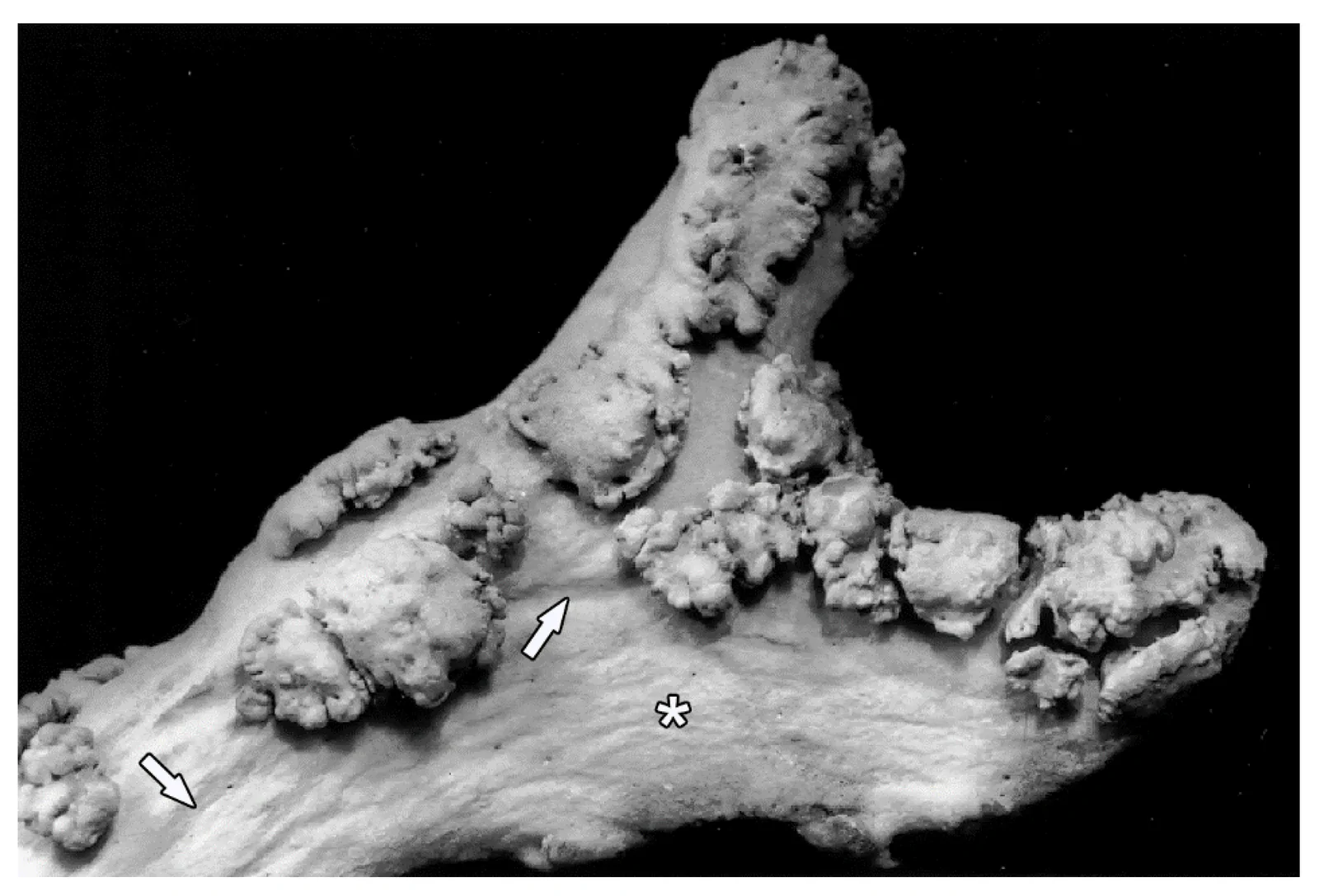
Surface of a macerated antler of a castrated fallow buck. Note numerous bony exostoses (with a coralloid surface structure) emanating from the original antler cortex (asterisk). Arrows: grooves on the antler surface marking the course of blood vessels, which had become partly overgrown by the newly formed bone.

Like their size, the composition of the abnormal structures formed in castrate antlers also varies widely. In the European roe deer, there is both massive bone apposition onto the original antler cortex and the formation of large skin outgrowths that can become pendulous (Figure 8b) [84,127,128,129,134]. Moreover, intradermal bone has been reported to occur in peruke antlers of this species [129,138], as well as in the antlers of a presumably hypogonadal male moose [131]. According to Goss [132,139], the nodular protuberances formed on the antlers of castrated fallow bucks are skin outgrowths, some of which can become pendulous during later development. These skin outgrowths, for which Goss [2] coined the term antleromas, are composed primarily of collagen fibers and fibroblasts, but contain no bone [132]. We have shown that, in addition to skin outgrowths, the antlers of castrated fallow bucks also develop conspicuous bony protuberances [133]. This matches previous findings by Richard Owen (1804–1892) who had already noted that the antlers of castrated fallow bucks “*become thickened by irregular tuberculate masses of bone*” ([126], p. 631). Such bone apposition was not mentioned by Goss [132] in his study on castrate antlers in fallow deer. Our study further demonstrated that the bony protuberances on the antlers of the castrated fallow bucks are formed by direct subperiosteal (intramembranous) apposition [133]. This apposition of new bone onto the original antler cortex started with the local formation of pedunculated exostoses that could later become confluent (Figure 9). Due to this process, blood vessels originally located at the antler surface could become overgrown.

In antlers of castrated reindeer, large fibromatous outgrowths of the velvet have been observed that resemble the skin outgrowths found in the antlers of castrates from other deer species [65]. Such structures have not been reported from female reindeer, but, unlike the situation in other cervid species, were rarely seen also in antlers of intact males [65].

In summary, there appears to be considerable interspecies variation in the formation and histological structure of the outgrowths developing on the antlers of castrated or hypogonadal male deer. In addition, there also seem to be certain intraspecific differences that may, among other things, depend on the stage of the antler cycle at which androgen withdrawal occurs. Future studies addressing the structure of castrate antlers are therefore warranted.

**Figure 10:**
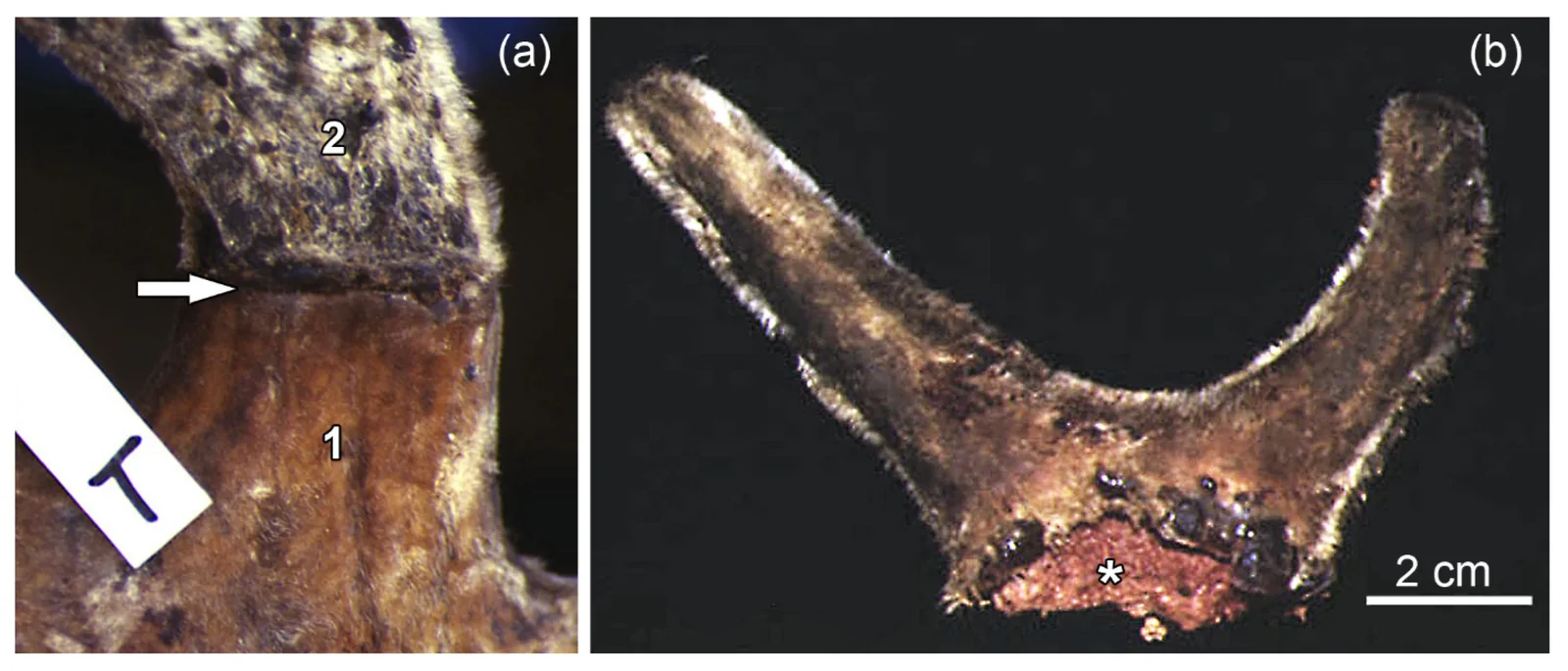
Effects of frostbite on the antlers of castrated fallow bucks (for details, see [124]). (**a**) Demarcation line (arrow) between the proximal living antler portion (1) and the necrotic distal one (2). Due to intense osteoclastic activity, the necrotic portion was subsequently detached along this line. Note that both antler portions are covered by velvet that, however, differs in appearance. (**b**) Sequestered distal antler portion of a further antler whose tip regions had become necrotic due to frostbite. Asterisk—separation plane of the necrotic antler portion.

When the permanently velvet-covered antlers of castrates are exposed to freezing temperatures for longer periods of time, their distal portions can die off from frostbite, followed by sequestration of the necrotic parts (still in velvet), wound healing and limited regrowth from the antler stumps (Figure 10) [123,124].

## 5. Developmental Origin of the Primary Cranial Appendages of Deer

The questions of which tissue is responsible for the formation of the primary cranial appendages (pedicles and first antlers) of deer, the derivation and specific properties of this tissue, and its interaction with other tissues have been in the focus of antler research over the past decades. Adam Olt (1866–1955) suggested that pedicles and first antlers (as well as the regenerated antlers) are derivatives of the skin overlying the prospective pedicle sites on the forehead. He stated that in deer “*two symmetrically arranged skin islands on the frontal bones behave as fetal tissue portions throughout life; these islands hold the growth potential for the antlers, whose development starts some time after birth and is recapitulated each year after antler casting. These skin islands are much more cell-rich than other skin and richly supplied with blood vessels and nerves. In the red deer, antler growth only starts about 7 months after birth, namely in the papillary layer and then in the corium. [….] The skin islands with the attributes of fetal tissue are now located as a seam—“formative seam”* [German: *“Bildungssaum”*]*—at the upper rim of the pedicles. From this formative seam, formation of the antlers proceeds annually distal to the pedicles. The antlers are therefore from their initial formation dermatogenic structures.*” ([129], p. 132; authors’ translation). If that were actually the case, the development of the pedicle and first antler in deer would correspond to horn initiation in bovids. In the latter, there is evidence that it is the connective tissue of the skin, not the subjacent periosteum, that is responsible for early horn development by inducing the formation of the bony horncore [140,141,142,143]. However, contrary to Olt’s suggestion, which apparently was not based on experimental findings, later studies clearly demonstrated that the skin overlying the prospective pedicle sites of deer is not responsible for initiating pedicle and first antler growth [144,145]. Thus, removal of the skin from this site and its replacement with skin from other areas of the forehead in juvenile male deer did not preclude the growth of cranial appendages, and the (autologous) transplantation of the skin from the prospective pedicle site to other body regions did not induce ectopic antler growth.

**Figure 11:**
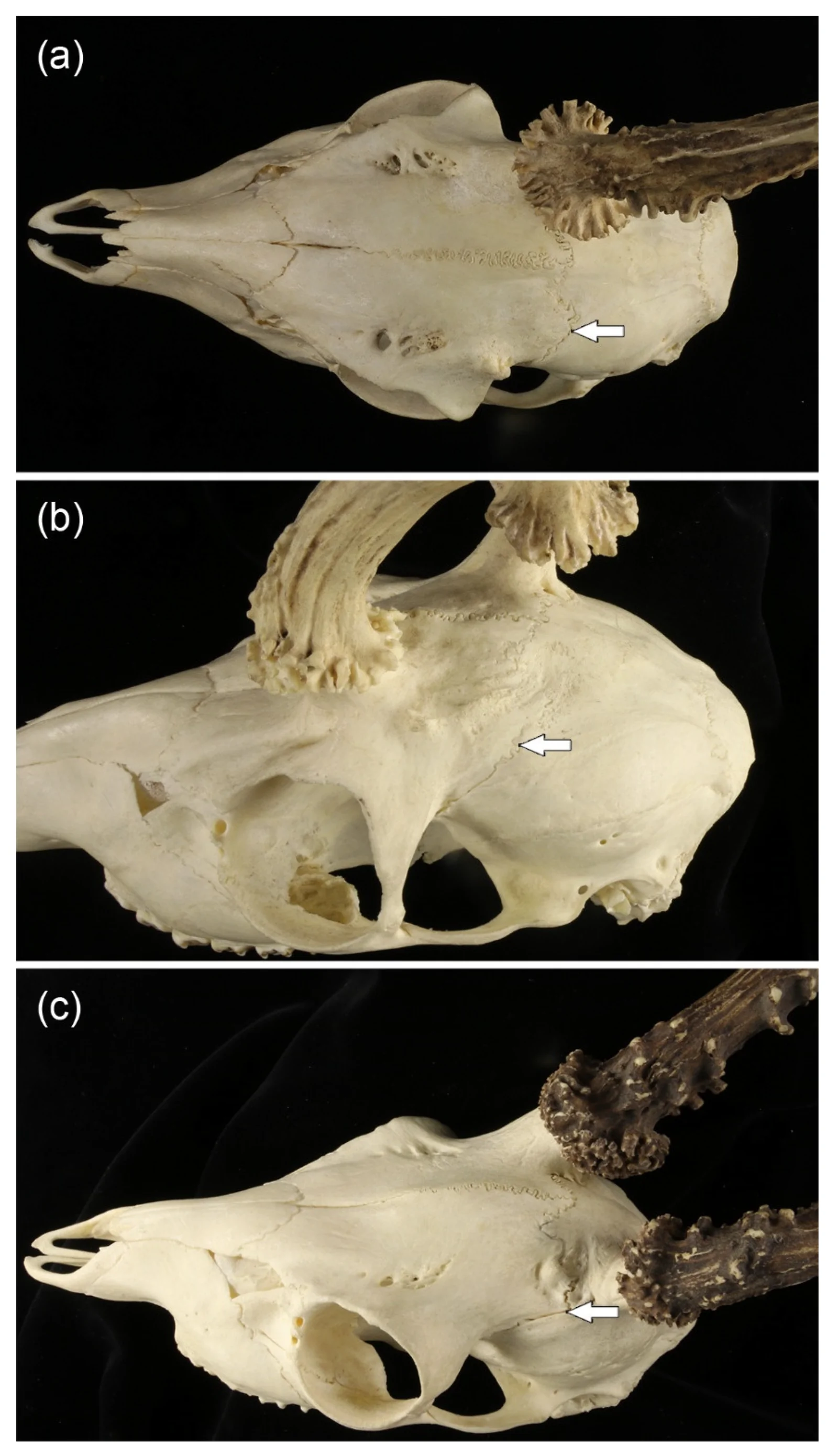
Crania of male European roe deer (*Capreolus capreolus*) from the experiments of Hermann Hartwig addressing the role of the periosteum overlying the future pedicle site (=AP) for cranial appendage formation in cervids. All interventions were performed on the left side of the cranium, while the right side served as a control. (**a**) AP-deletion prevents cranial appendage formation. (**b**) Forward shifting of the AP causes a more anterior location of the cranial appendage on the frontal bone. (**c**) Shifting of the AP to a more posterior location causes cranial appendage formation on the parietal bone. All antlers are regenerated ones. The arrows mark the frontoparietal (coronal) suture.

**Figure 12:**
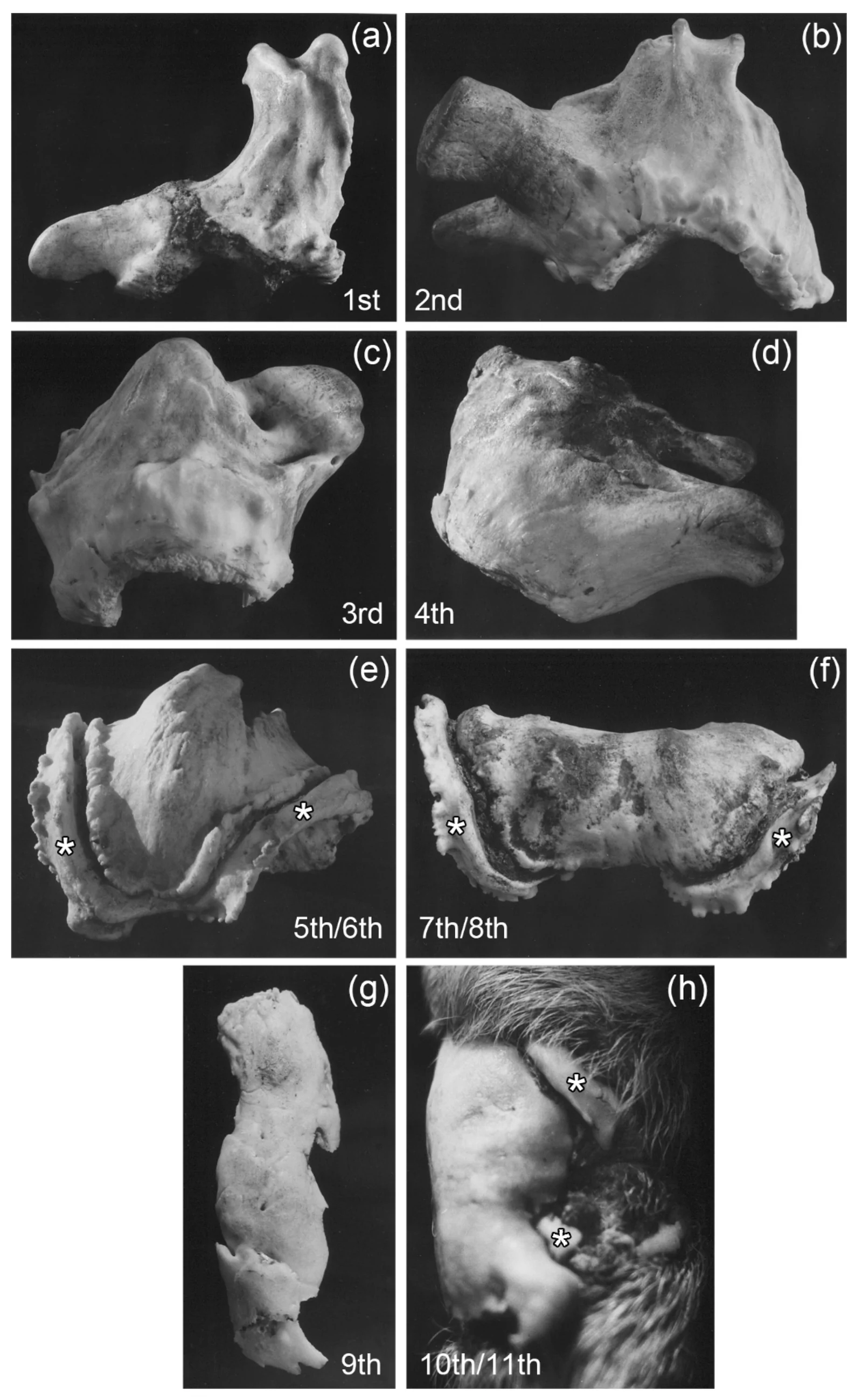
Ectopic antler structures (**a**–**h**) grown during consecutive years at the foreleg of a European roe buck (*Capreolus capreolus*) following autologous transplantation of AP to the metacarpal region [146]. The first four ectopically grown antlers (**a**–**d**) were cast around the normal time for the species. In contrast, the 5th, 7th and 10th antlers were not cast, causing the repeated formation of “double-head antlers” (**e**,**f**,**h**), the last of which (**h**) was still attached to the foreleg when the buck died at the age of 12 years. The second antler generation of the double-head structures is marked by asterisks. Repeated double-head formation was apparently caused by the broad connection of the ectopic antler structure with the underlying metacarpal bone due to the lack of a pedicle structure. This broad connection could not be fully resorbed prior to the onset of antler regeneration. Note that, like in the double-head antlers shown in Figure 5, the two successively produced antler structures are separated by a furrow, originally occupied by the velvet covering the second antler generation. Also note the progressive morphological simplification with age of the ectopic antler formations to plate-like structures. The “antler nature” of these bone formations was proven by the fact that they were covered by velvet that was lost from the structures at the normal time for the species. Images not to scale. Photos courtesy of the late Hermann Hartwig.

In contrast, as was first shown by the pioneering experiments of Hermann Hartwig (1910–2012) on male European roe deer fawns, conducted during the 1960s and early 1970s, formation of the primary cranial appendages depends on the periosteum overlying the prospective pedicle site [145,146]. Removal of this specialized periosteum, for which Goss [2] later coined the term antlerogenic periosteum (AP), prevented cranial appendage formation, while its autologous transplantation (in the form of a pedicled flap) to other cranial sites caused ectopic pedicle and antler growth (Figure 11). In these experiments, the transplanted AP was always inserted between the autochthonous periosteum and the underlying bone [145,146]. Finally, it was demonstrated that following free transplantation of the AP to the metacarpal region of a juvenile roe buck, antler growth was also initiated at this remote graft site [146]. The complete sequence of the consecutively formed antler structures at the foreleg of this experimental animal is (for the first time) shown in Figure 12. The findings on the decisive role of the AP for pedicle and first antler formation constitute a major breakthrough in the history of antler research and were the starting point for numerous studies analyzing the properties of the AP and its interaction with other tissues.

**Figure 13:**
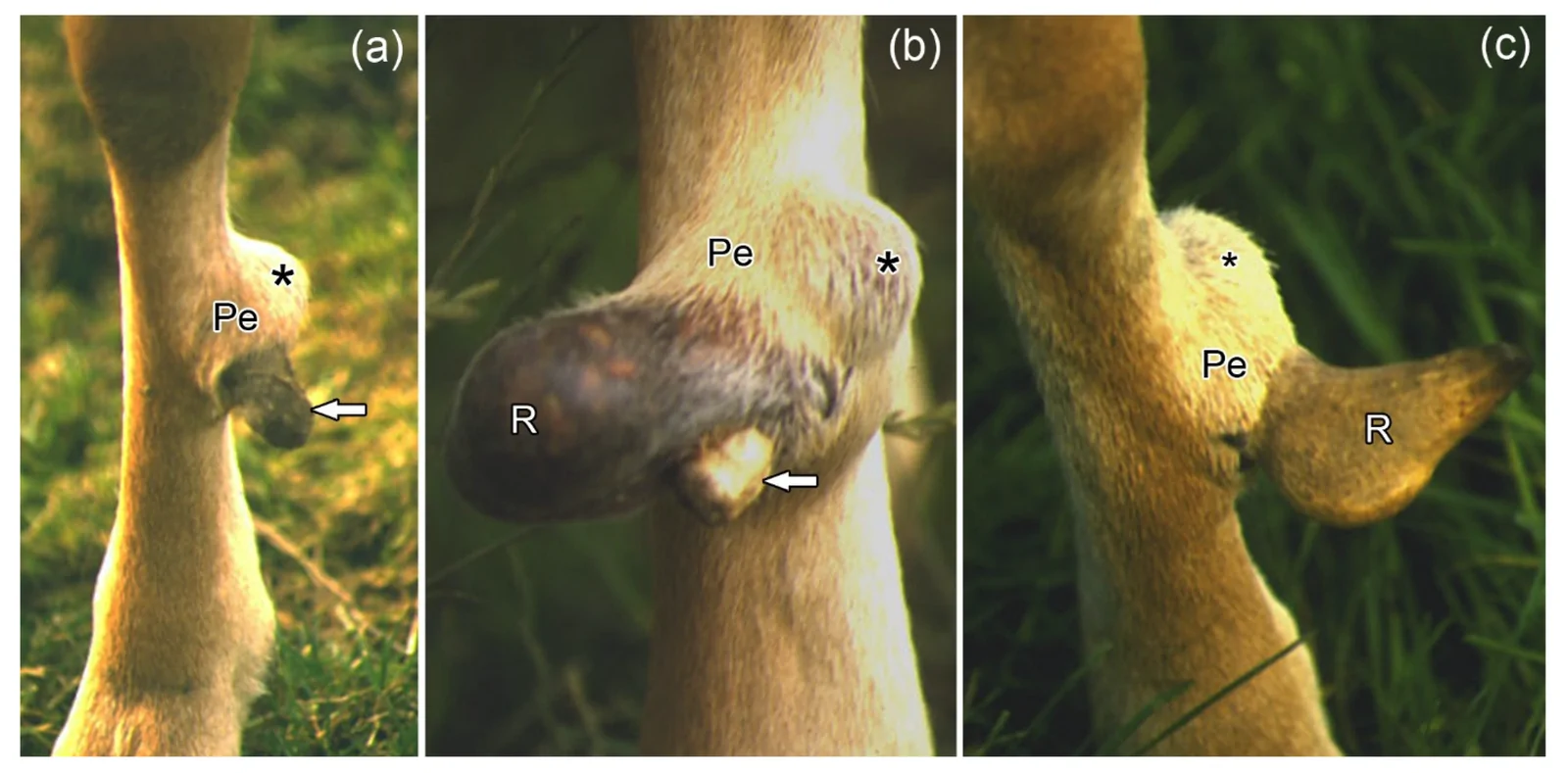
Ectopic antler growth in the metacarpal region of a fallow buck (*Dama dama*) following autologous (subcutaneous) transplantation of AP. Pe: pedicle covered by normal leg skin, asterisk: posterior outgrowth from pedicle likewise covered by normal leg skin. (**a**) First antler (arrow) still covered by (dark) velvet. (**b**) Regenerating antler (R) covered by (dark) velvet, and undetached hard first antler (arrow). (**c**) Regenerated antler (R) from which the velvet has been lost. For details of the experiment (see [148]).

The findings of Hermann Harwig in the European roe deer [145,146] were later corroborated in experiments on other deer species (Figure 13) [67,147,148,149]. As is indicated by the results of the transplantation experiments, the AP cells are committed to a specific differentiation pathway and the AP is capable of autonomous differentiation [19,150].

The AP, which is composed of different cell populations [151], is considerably thicker than the surrounding frontal periosteum [51,152]. The cells of the AP’s inner cellular (cambium) layer are rich in glycogen, which is regarded as an energy source for rapid proliferation [153].

As is demonstrated by the occasional occurrence of antlered females in deer species other than *Rangifer tarandus* [154,155,156,157], the specialized AP, which is present in both sexes, can be stimulated to initiate pedicle and antler growth also in females. Normally, the AP is not activated in females due to the lack of an appropriate endocrine stimulus. One possibility for triggering pedicle and antler growth in female deer is the presence of an androgen-producing tumor [154,156]. Experimentally, pedicle and subsequent antler growth could be induced in ovariectomized female white-tailed deer [123] and in one of three intact female red deer [158] by administration of testosterone. In the other two intact females treated with testosterone, only pedicles but no antlers developed [158], matching previous observations by Jaczewski [159] on intact and ovariectomized red deer hinds. In the latter case, onset of antler growth could be triggered by partial pedicle resection [159]. In addition to a hormonal stimulus, the AP of female cervids can also be activated to start pedicle growth by unspecific (traumatic) stimuli (e.g., [160]).

Experiments by Goss [161] further demonstrated that cells of the AP also contain information for the axial orientation of the antlers. Thus, in situ rotation of the AP by 180 degrees caused the formation of primary as well as regenerated antlers with a reversed orientation of their anterior–posterior axis. Other authors later suggested that the morphogenetic information of the AP is primarily held in its anterior-medial portion [162].

Induction of ectopic antler growth by subcutaneous transplantation of AP shows that the skin from many body regions has the competence for velvet transformation and thereby participation in antler growth. The capacity of the skin to react to the subcutaneous formation of a pedicle structure with velvet transformation was systematically explored by Goss [163]. He found that the skin on the fore and hind legs, hip, forehead and ear can participate in antler formation by velvet transformation, while the hairless skin of the rhinarium and ventral tail surface as well as the sparsely haired back skin overlying the spine cannot. A small antler structure was even formed when the AP was transplanted to the anterior chamber of the eye, with velvet being formed by the cornea [164]. While subcutaneous grafting of AP resulted in ectopic antler growth, intramuscular transplantation of AP into the shoulder region only caused the formation of a bony nodule, but no antler structure [164]. Based on his findings, Goss [163,164] reasoned that a prerequisite for antler growth is a close contact between AP-derivatives and the overlying skin, caused by the expansion of the bony pedicle, that allows inductive interactions between the two components.

The concept of Goss [163,164] was later corroborated by experimental findings of Chunyi Li and colleagues. Thus, insertion of an impermeable membrane between AP and overlying skin prevented antler growth, while insertion of a permeable membrane did not. This suggested that the inductive action leading to velvet transformation and, in consequence, antler formation is caused by diffusible molecules rather than a direct physical contact between the interacting tissues [165]. The experiment further showed that the required interaction can be of a transient nature, as insertion of an impermeable membrane after onset of first antler formation did not stop antler growth. Based on the findings of a further study using a nude mouse model, it was suggested that an inductive signal from the AP acts on the overlying dermis, leading to a transformation of the epidermis via paracrine and juxtacrine mechanisms. It was further proposed that the transformed epidermal cells then produce a feedback signal that initiates the transition from pedicle to first antler growth [166].

**Figure 14:**
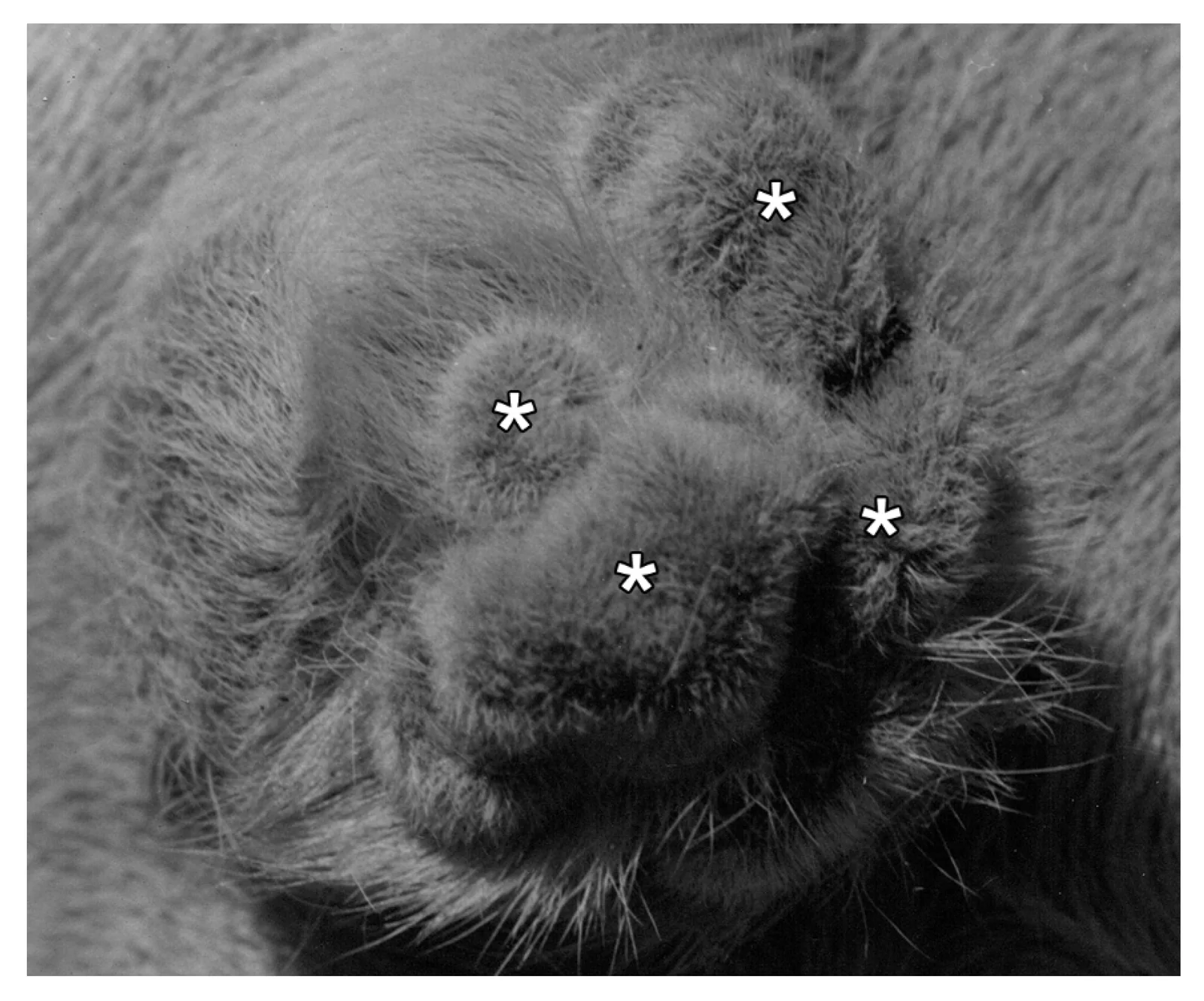
Localized velvet transformation of the skin (asterisks) and start of antler formation from the (belatedly formed) pedicle of a fallow buck.

Interestingly, velvet transformation and start of antler growth can be a localized event that affects only certain areas of a pedicle, as was observed in belatedly formed pedicle structures that had developed in a fallow buck after incomplete AP removal (Figure 14) [19]. In this case, probably only certain cells of the periosteum/perichondrium covering the pedicle released the inductive signal that caused velvet transformation of the overlying skin and led to localized antler formation.

Using a tissue inversion approach, it was shown that the cell-rich cambium layer of the AP (as opposed to the fibrous layer) is the likely source of the inductive signal acting on the overlying skin [149]. Thus, inversion of the AP (cambium layer facing the skin) caused an earlier onset of velvet transformation compared to normally oriented AP (cambium layer facing the bone).

In vitro, AP cells express typical markers of mesenchymal stem cells and can differentiate into chondrocytes, osteocytes and adipocytes, thereby demonstrating their multipotency [167]. In the AP of male and female sika deer (*Cervus nippon*), recently a sub-population of THY^+^ mesenchymal stem cells (MSCs) has been identified that highly expresses both the androgen receptor (AR) and the relaxin/insulin-like family peptide receptor 2 (RXFP2) [168]. Expression of *Hox*-genes was reduced in these cells, suggesting a high developmental plasticity. It had previously been shown that in vitro treatment of AP cells with dihydrotestosterone upregulates RXFP2 expression [151], which is in line with the established view that AP activation is caused by an androgen surge. The study by Ba et al. [168] also provided evidence that canonical Wnt signaling is involved in the activation and differentiation of the RXFP2^+^ AP-MSCs.

It was further demonstrated that the initiation of pedicle and antler growth from the androgen-activated RXFP2^+^ AP-MSCs of male sika deer involves the participation of M2 macrophages. Recruitment of the macrophages is promoted through interleukin-34 (IL-34) secretion by the AP-MSCs [168]. Macrophage depletion inhibited antler growth in male deer, and prevented the formation of bony outgrowths (‘antlers’) in AP-engrafted nude mice. Interestingly, IL-34 was not released in the AP of female sika deer; however, pedicle and subsequent antler growth could be induced by injection of autologous macrophages, the macrophage chemokine CCL2, or the immune response inducer lipopolysaccharide into the presumptive pedicle site of the females [169].

In the reserve mesenchyme present at the tip of growing antlers and in the pedicle periosteum (PP) responsible for antler regeneration (see below), RXFP2 expression was significantly down-regulated compared to the AP, while in 17 other analyzed deer tissues, including frontal periosteum from outside the pedicle anlage area, no RXFP2 expression was detected [168]. Moreover, while high-level expression of RXFP2 was also observed in the AP of other antlered cervid species (wapiti, *Cervus canadensis*, and *Rangifer tarandus*), this was not the case in the frontal periosteum present in the same location in the antlerless water deer. This indicates that RXFP2 is specifically expressed in the AP of antlered deer [168]. Pseudogenization of the *RXFP2* gene in *Hydropotes inermis* (and in Moschidae) had previously been reported by Wang et al. [18].

Comparison of RXFP2^+^ AP-MSCs with bone marrow-derived MSCs showed that the two groups differ in the expression of mesenchymal stem cell markers. RXFP2^+^ AP-MSCs highly expressed markers indicative of their origin from the cranial neural crest and transcription factors related to early osteogenic and chondrogenic differentiation, while adipogenic markers, which were highly expressed in bone marrow-derived MSCs, were not expressed in the AP stem cells [168]. Based on these findings, the preferential use of neural-crest derived cell material for the repair of bone defects in skull elements of neural crest origin was recommended by Ba et al. [168].

## 6. Tissue Origin of the Regenerating Antler and the Nature of the Antler Blastema

The replacement of the cast antlers by a pair of new ones is a remarkable example of epimorphic regeneration, i.e., the regrowth of a lost structure from an anatomically complex stump [170]. As antler regeneration occurs regularly over the lifespan of an animal, it may be classified as a case of extensive physiological regeneration [45,47,48]. However, antler regrowth also qualifies as an example of reparative regeneration, as it is caused by damage to a complex organ (at velvet shedding), followed by the death and regrowth of this organ [44,47]. This restitution involves a wound healing process and the formation of a regeneration blastema.

**Figure 15:**
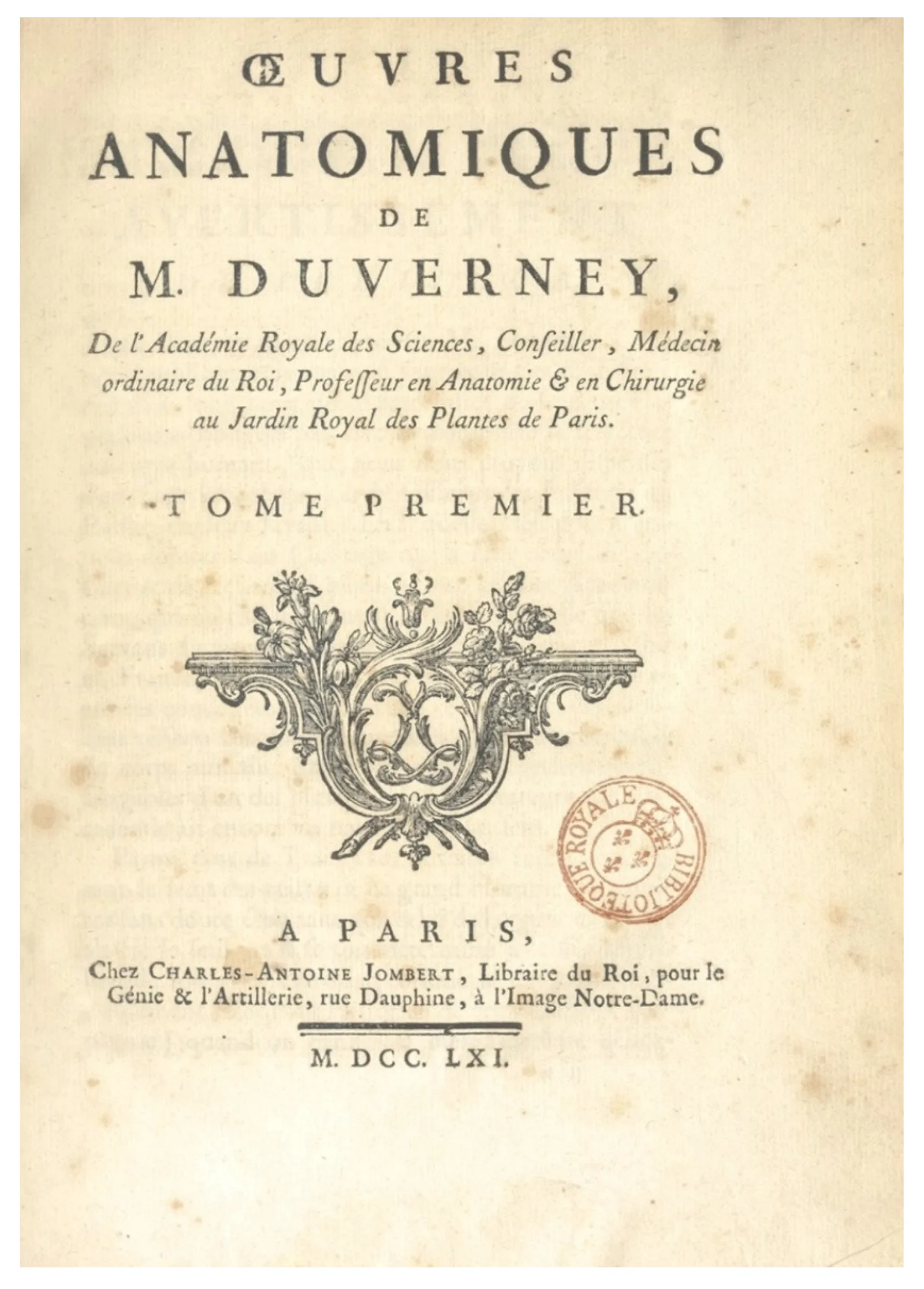
Title page of volume 1 of the *Œuvres Anatomiques de M. Duverney (1761)* [172], in which Duverney expresses a preformationist view of antler regeneration. Source: Bibliothèque nationale de France.

It is evident that the capacity for periodic antler regeneration must reside in the pedicle from which the regrowth process starts. However, the question of the precise origin of the regenerative tissue forming the new antler has been answered differently over the last centuries. In volume one of his posthumously published *Œuvres Anatomiques* (1761) (Figure 15), Joseph-Guichard Duverney (1648–1730) [171] stated the following: “*I think one can believe that in these apophyses of the forehead bone, named crowns* [i.e., the pedicles] *there are kinds of germs for the new production of antlers that successively come into existence each year. Although all these small antlers are indiscernible owing to their very minute size, they are nonetheless able to receive nutrients and grow […], and if some are sprouting rather than others, this is only due to their particular arrangement*.” ([172], p. 349; authors’ translation).

The view on the mechanism of antler regrowth expressed by Duverney is the typical explanation for regeneration given by proponents of preformationism, an embryological theory that was popular in the 17th and 18th centuries [173,174,175,176]. Preformationism posited that organisms develop from miniature versions of themselves present in either sperm or ovum, and during ontogeny just “unfold” and grow in size. This idea was combined with the view that all future members of a species had been formed simultaneously by God at creation and were encased within one another in the gonads of the first ancestor of that species to sequentially start unfolding in successive generations ([174,175,176,177]. In contrast, the rivaling theory of epigenesis held that organisms gradually develop by stepwise differentiation from initially unorganized matter [175,176].

To explain the phenomenon of regeneration within the framework of preformationist thinking, advocates of this theory claimed the presence of preformed germs for the repair of lost parts in the intact organism (for a discussion, see [173]). The explanation of antler regrowth given by Duverney (1761) [172] clearly reflects this view. The “particular arrangement” of the antler germs mentioned by Duverney presumably relates to the fact that the successively formed antlers in many deer species (e.g., red deer) tend to increase in size and complexity. To achieve this, the “antler germs” must be arranged and start their development in a pre-determined order. The theory of preformation and encasement increasingly lost acceptance from the mid-18th century onwards [173].

On the basis of morphological observations, Berthold [50] concluded that the regenerating antler is derived from the pedicle skin. As previously mentioned in the paragraph on primary cranial appendage formation, Olt [129] likewise posited that the regenerating antler is a dermatogenic structure derived from the so-called “*Bildungssaum*”, i.e., the rim of skin covering the distal pedicle. Based on histological observations, George B. Wislocki later stated that “*one gains the impression that it is the proliferating deeper portion of the corium, rather than adjacent periosteal tissue, which restores the surface of the pedicle and gives rise to the osteogenic germinal bed. Thus we are led to postulate that the growth of the entire antler is probably dependent upon and controlled by the formation of a germinal or periosteal bed derived from the skin*.” ([96], p. 379). This view was initially shared by Goss ([43,178], who concluded the following: “*The source of the cells that make up the antler bud has not been precisely identified, but the dermis is almost certainly the major contributor*.” ([43], p. 570). Later, however, he adopted a more cautious position and argued that, comparable to other regenerating systems, “*it is probable that most of the tissue types in the pedicle contribute cells to the antler bud*” ([179], p. 233) and that “*it would not seem unreasonable that the various component tissues in the pedicle stump might possibly give rise to their counterparts in the antler, all somehow responding simultaneously to a common stimulus*” ([139], p. 295).

The view that the skin rim covering the distal pedicle is the tissue source of the regenerating antler was rejected by Bubenik and Pavlansky [83], based on the observation that antler regrowth can take place even when the regenerative skin rim (*Bildungssaum*) had been surgically removed from the pedicle or been replaced by skin from the parietal region. A later study by Li et al. [180] showed that antler regeneration from pedicle stumps can occur when the skin is prevented from participating in the process, thereby leading to the formation of (stunted) “skinless” antlers. These findings exclude the pedicle skin as the tissue source for the regenerating antler.

Different studies have instead demonstrated that the actual source of the cells that form the regenerating antler is the PP, which is a derivative of the AP responsible for the formation of the primary cranial appendages. This view was initially based on the analysis of double-head antlers, which provided evidence that the second antler generation of this structure develops as a periosteal exostosis from the distal pedicle [115,116,117]. The view that the PP is the cell source for antler regeneration is supported by histological observations showing that the periosteum of the distal pedicle is thickened prior to the onset of antler regeneration [86,110], and that in red deer the anterior and posterior growth centers of the regenerating antler are formed by local thickenings of the PP already before the completion of wound closure at the top of the pedicle stump [86]. Finally, it was shown that PP deletion prevented antler regeneration during the following antler growth season or delayed this process until after the PP had regenerated [181].

It has been suggested that the PP cells responsible for antler regeneration are unable to initiate pedicle and first antler growth and that their developmental potential is therefore restricted compared with that of the AP [182]. This was based on the observation that transplantation of a mass of PP twice to three times larger than the minimum mass of AP needed to induce ectopic pedicle and antler growth failed to initiate this process. As mentioned previously, like the AP cells also the PP cells and the cells of the reserve mesenchyme, the outermost layer of the antler growth zone, express surface markers of mesenchymal stem cells and can be induced to form osteocytes, chondrocytes and adipocytes in vitro [167,183].

Following antler casting, there is some bleeding from the bone wound formed at the top of the pedicle (Figure 2a), followed by the formation of a blot clot, which upon drying forms a scab covering the casting surface (Figure 2b). This is followed by the ingrowth of the epidermis (followed by cells from the dermis) over the wound area that starts from a tumescent rim of distal pedicle skin that forms already prior to casting [47,86,110]. A layer of granulation tissue has been reported to overlie the exposed bone of the pedicle stump [86,106,110,184], and it was suggested that this tissue contributes to the developing antler bud [106]. This view was considered unlikely by Kierdorf et al. [47], who instead proposed that the granulation tissue is replaced by mesenchymal tissue during formation of the regeneration blastema.

Skin wounds in adult mammals heal by a fibrotic process that involves a strong inflammatory response and leads to the formation of a scar, a structure rich in extracellular matrix and devoid of cutaneous appendages [185,186]. Scarring involves the transdifferentiation of fibroblasts to myofibroblast that cause wound contraction and act as the main source of the excessively produced collagen-rich extracellular matrix [186,187]. The dysregulated tissue repair leading to scarring constitutes a major clinical challenge, and there is a strong need for finding ways to reduce scarring and promote scar-free skin regeneration [186,188]. Notably, the wound healing process over the apex of the pedicle stump that occurs after antler casting involves no or only minimal scarring [8,47,139,186,189]. Such essentially scar-free (regenerative) wound healing is also seen in other cases of appendage regeneration in vertebrates [47,186]. In reindeer, it was shown that velvet fibroblasts possess properties of fetal fibroblasts, which limits inflammatory responses, whereas back skin fibroblasts express inflammatory mediators similar to pro-fibrotic adult human and rodent fibroblasts [189].

Regenerative cutaneous wound healing over the pedicle stump involves both local and systemic factors [8,190,191,192]. Regarding the influence of local factors, it was shown that the injection of stem cells isolated from the antler regeneration blastema or of exosomes formed by these cells around full-thickness excisional (FTE) skin wounds of rats improved the quality of the healing process compared to injection of exosomes originating from bone-marrow derived MSCs. As was further demonstrated, administration of exosomes derived from antler stem cells effectively inhibited the transdifferentiation of fibroblasts to myofibroblasts [190]. Recently it was demonstrated that exosomes derived from antler stem cells also promoted the regenerative healing of severe burn wounds in rats [191].

Another study demonstrated that the healing mode of FTE skin wounds on the forehead of sika deer depended on the time of wounding in relation to the antler cycle [192]. While wounding during the period of antler regeneration (ARP) led to regenerative wound healing, wounding outside of this period, i.e., during the hard antler period (non-ARP), resulted in scar formation. Subsequently it was shown that topical application of blood plasma obtained from deer during the ARP to FTE skin wounds of rats promoted regenerative healing, while plasma obtained during the non-ARP did not. The positive effects of the treatment with ARP plasma included increased cell proliferation, a reduced inflammatory response, reduced myofibroblast formation, an increase in the TGF-β3 to TGF-β1 ratio, and an enhanced extracellular matrix regeneration. Comparative proteomics indicated upregulated factors (such as IGF-1 and proteoglycan 4), with a potential to stimulate regeneration, in the ARP plasma. Topical application of either IGF-1 or of IGF-1 plus proteoglycan 4 promoted skin wound healing in rats, with the combined application being more effective. These results indicate a significant role of systemic factors (present in deer blood obtained during the ARP) in regenerative wound healing [192].

The fact that the observed effects of the local and systemic factors in the wound healing processes were not species-specific, may lead to novel approaches for improving regenerative wound healing in the clinical setting.

Immediately after antler casting, ongoing osteoclastic activity in the distal pedicle causes a smoothing of the uneven casting surface [70,110]. This is followed by osteoblastic activity that initially leads to a thickening of the bony trabeculae of the distal pedicle. Subsequently, a system of slender bony trabeculae is formed that extends from the thicker trabeculae of the pedicle stump. This causes a partial restoration of the pedicle portion lost along with the detached antler [70,96,110]. The formation of this narrow zone of slender trabeculae during early pedicle restoration occurs by intramembranous ossification [70,110].

**Figure 16:**
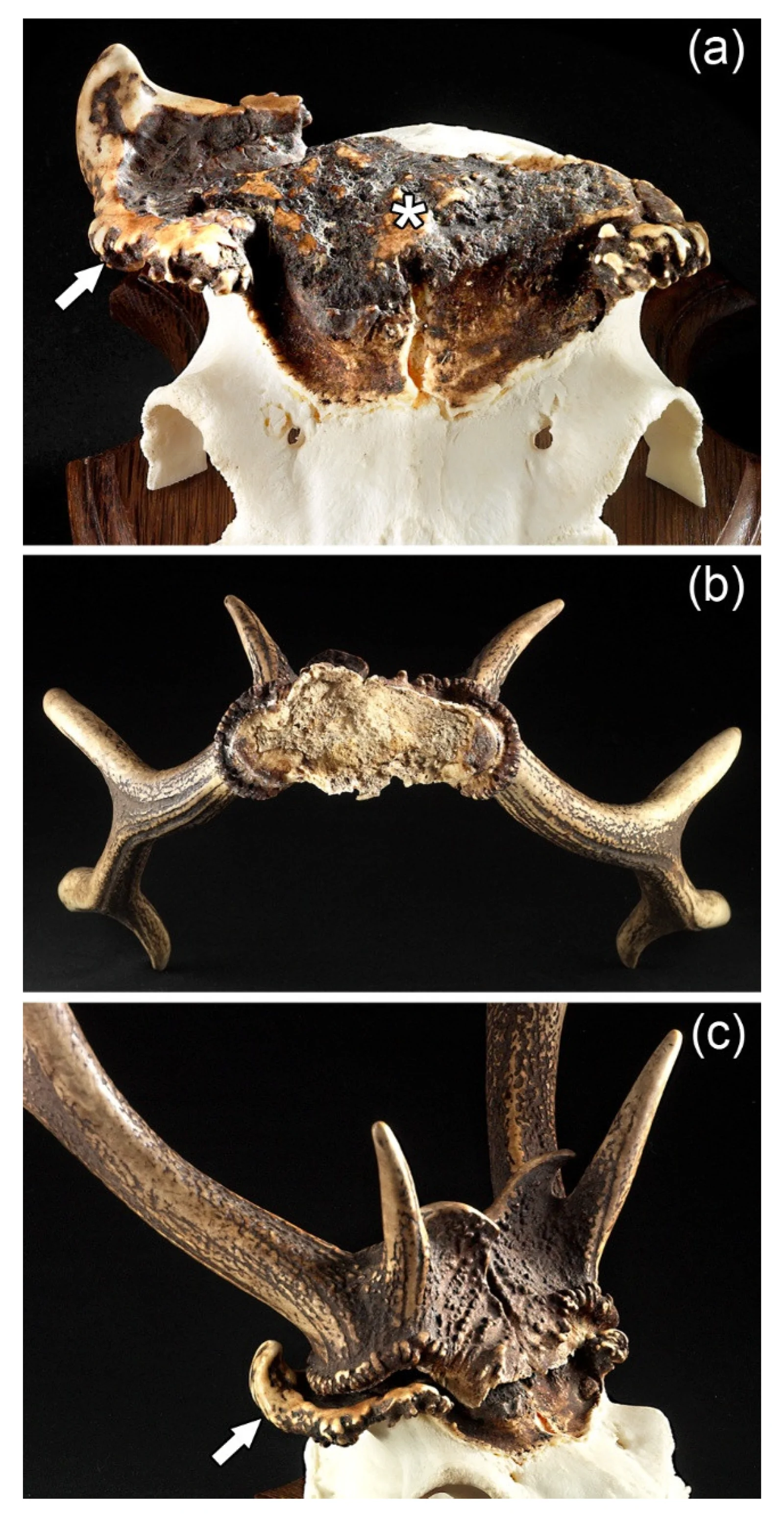
Pedicle and antler fusion, and formation of a double-head antler in a 12–14 yr-old sika stag (*Cervus nippon*). For details of the case, see [194]. (**a**) Abnormal antler structure present in the stag when it was shot. Asterisk—separation surface of the fused pedicles, arrow—newly formed antler bone (second antler generation of the double-head structure). (**b**) Basal view of the belatedly cast, basally fused antlers. (**c**) Belatedly cast, basally fused antlers refitted to the skull. Arrow—newly formed antler bone.

The loss of the distal pedicle portion occurring at antler casting is not fully compensated afterwards, and the pedicles therefore progressively decrease in length with age [50,108,193]. In consequence, in old animals with much shortened pedicles, the separation planes formed at antler casting may be located close to the level of the skull roof. In such cases, the normally separated resorption areas of the two pedicle remnants can become confluent. Figure 16 shows the case of an old sika stag, in which this had likely occurred and new bone growth had led to the fusion of the pedicle remnants and also of the basal antler portions [194]. This resulted in an abnormally broad connection of the regenerated antlers with the skull. During the following antler cycle, this bony connection was not sufficiently resorbed to allow for casting prior to the onset of antler regrowth. In consequence, a double-head antler was formed. Belated casting of the old, basally fused antlers then exposed a single broad separation surface that was laterally rimmed by the newly produced antler bone. The findings in the sika stag illustrate some important biological functions of the pedicles, which are (1) to provide a connection between antler and skull roof of a diameter that can be eroded during the limited timespan of osteoclastic activity in the pedicle, and (2) to keep the two fracture planes formed at antler casting separate from each other and at a “safe” distance from the skull roof.

The initial pedicle restoration phase is followed by the establishment of a regenerative antler bud. There has been some discussion about whether the antler bud can be classified as a blastema, the formation of which is a hallmark of epimorphic regeneration [44]. Applying the definition of a blastema as “*a heterogeneous cell mass that through migration and proliferation transiently forms at the injury site and undergoes morphogenesis to form the missing organ*” ([48], p.191]), the regenerating antler bud can certainly be classified as a type of blastema. The antler blastema, however, differs in several important aspects from the “classic” regeneration blastema, which is formed for instance during limb regeneration in urodeles [195], or following amputation through the distal phalanx of mice [48,196]. Thus, for instance there is no extended histolysis and no apparent dedifferentiation of cells in the pedicle stump, and the antler blastema is populated by mesenchymal stem cells derived from a single source, the PP. For further discussion of the nature of the antler blastema, see [44,47,107].

## 7. Growth Rate, Blood Supply and Histogenesis of Antlers

Once the processes of wound healing and antler bud establishment are completed, the antlers start elongating and, in species with muti-tined antlers, undergo repeated (dichotomous) branching. The velocity of antler growth by far exceeds that of other bones and constitutes one of the exceptional features of antlers [2]. Antler elongation follows a sigmoidal curve, being highest during mid-growth [178]. Thus, for wapiti, a maximum elongation rate of 2.75 cm per day during peak antler growth has been recorded [178]. The antler growth rate must have been even higher in the extinct Pleistocene/early Holocene giant deer (*Megaloceros giganteus*) [197], which was first described by Blumenbach [198] as *Alces gigantea*. The weight of a pair of giant deer antlers could reach up to 40 kg [199], and the largest recorded antler span for the subspecies *M. g. giganteus* (holotype from Dunleer, Ireland) is given as 4.26 m [200].

**Figure 17:**
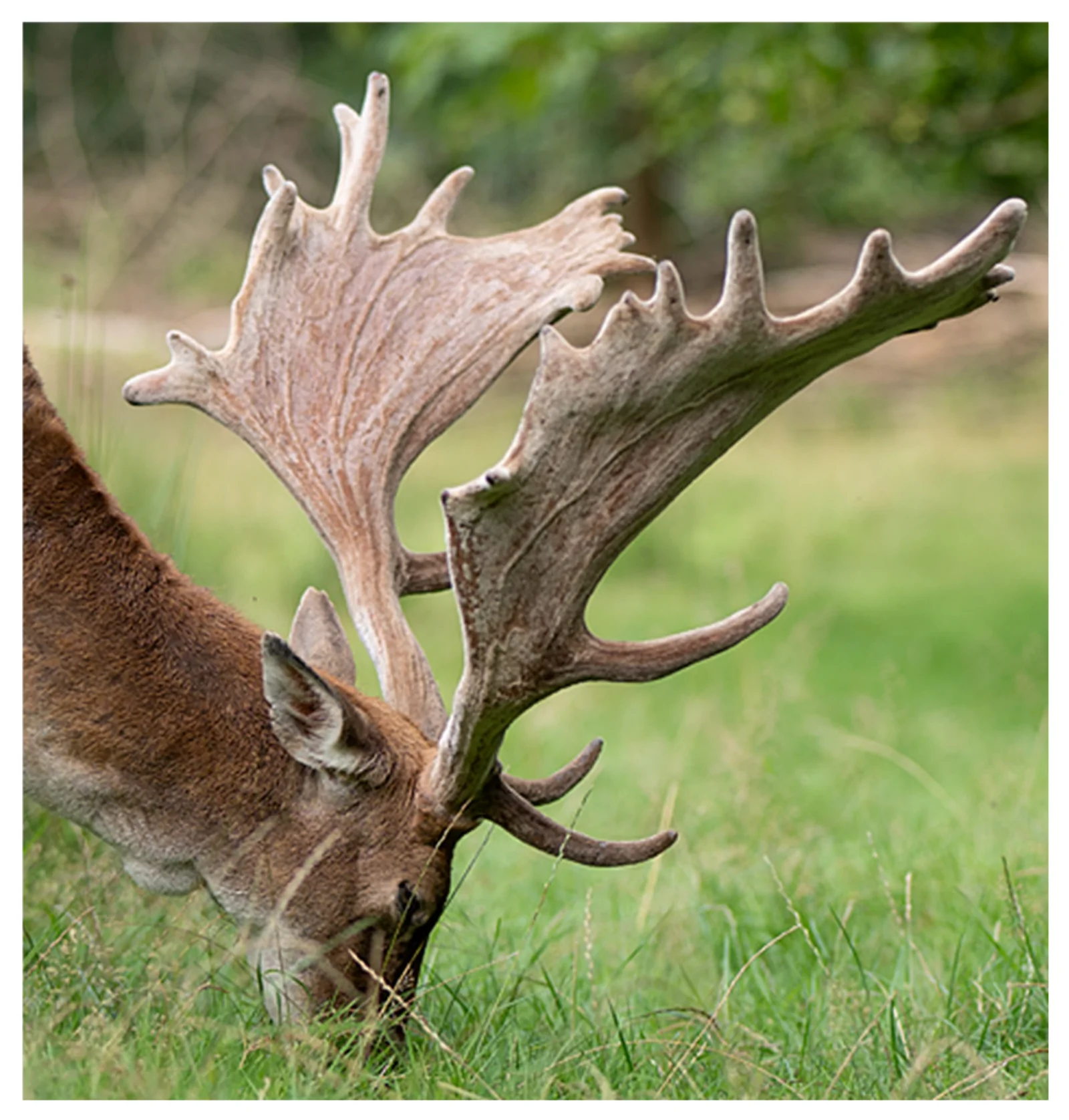
Velvet antlers of a fallow buck with well-visible course of the blood vessels located in the vascular layer of the velvet.

The enormous growth rate of antlers requires a copious blood supply to provide the necessary nutrients and oxygen needed for this process. Each antler is supplied by several arteries that arise from the superficial temporal artery and ascend in the vascular layer at the base of the velvet [201,202,203]. These arteries are thick-walled, possess a high resistance to compression and can rapidly close by constriction when severed [204]. According to Wislocki [205], antler arteries therefore structurally quite closely resemble umbilical arteries. Numerous arterioles branch from the ascending arteries along the antler shaft, and the antler growth tips are supplied by a system of capillary retia [201,202,203] that was already observed by Berthold [50]. During early stages of antler growth, venous drainage occurs mainly through veins connecting the interior portions of the antler and pedicle. However, due to the progressively dense ossification of the antler base (see Figure 5), during larger stages of antler growth, the blood return from the antlers largely or entirely takes place via venous collecting systems that drain into the veins of the vascular layer of the velvet [203]. The course of the blood vessels in the vascular layer is clearly visible upon external inspection of velvet antlers (Figure 17). In hard antlers, the former course of the larger vessels is visible as a system of grooves on the antler surface (Figure 9). Based on his injection experiments, Rhumbler [202] stated that antler arteries and veins are located in separate grooves.

Antlers require huge amounts of calcium and phosphorus for mineralization during their short growth period of only a few months. It has been calculated that antlers of giant deer with a dry weight of 40 kg would have contained 7.6 kg of calcium and 3.8 kg of phosphorus [199]. Calcium deposition in growing red deer antlers peaked between days 91 and 112 [206], and the simultaneous mineralizing surface area was highest between days 84 and 110 of antler growth [103]. It has recently been demonstrated that concomitant with antler bone formation there is also bone formation in the pedicles, leading to their compaction [107]. Other studies revealed marked variation in antler bone density and mineral content as well as mineral composition between basal and distal antler portions as well as a strong effect of nutrition on mineral profiles along the antler shaft (reviewed in [3]).

During antler growth, considerable bone resorption occurs in parts of the postcranial skeleton of male deer, which helps to supply the mineral elements needed for antler mineralization [207,208,209,210,211]. It has been calculated that more than half of the calcium requirement of red deer stags during the antler growth phase is met by mobilization from the skeleton [212]. The resorption spaces caused by this mineral mobilization process are refilled with new bone following cessation of antler growth or partly already before completion of this process [208,210]. This cyclic shift between bone resorption and repletion in the postcranial skeleton of deer has been referred to as “cyclic bone remodeling” [209], “cyclic physiological osteoporosis” [208] or “reversible osteoporosis” [210].

In a recent review, Li et al. [13] emphasized that the study of the seasonal bone resorption and repletion observed in male deer in relation to the antler cycle may provide novel insights for the treatment of osteoporosis in humans. Osteoporosis is a systemic metabolic bone disease characterized by abnormally low bone mass and microarchitectural deterioration of bone tissue with a consequent increase in bone fragility and susceptibility to fracture [213]. It can be asked whether the term “osteoporosis” should be applied to the resorptive changes in the postcranial skeleton of deer occurring during antler growth. The statement that the skeleton of male deer “*suffers severe osteoporosis annually and then fully recovers*” ([13], p. 2) is considered problematic, as there is currently no evidence that the seasonal bone resorption in the postcranial skeleton of deer is associated with an increased fracture incidence. Given this apparent lack of morbidity, it might be advisable to replace the term “osteoporosis” with “osteopenia” when referring to the reduction in in the density of some skeletal elements of deer during antler growth.

The histogenesis of antlers has been an issue of debate for long. In his monumental *Histoire Naturelle*, Georges Louis Marie Leclerc, comte de Buffon (1707–1788) reasons that the “*horns of the stag in their make and growth resemble the branches of a tree; and its substance is perhaps more of the nature of wood than bone*” ([214], pp. 46–47). He emphasizes the similarities in growth between trees and antlers by stating that “*nothing can bear a stronger resemblance to their* [the antlers’] *growth than that of a branch of tree; they extend from one extremity only, they are at first as tender as an herb and then harden like wood. The scurf which covers and grows with them is their bark, which the animals rub off when arrived at their full growth; until this is completed the ends remain soft, and likewise divide themselves into a number of branches*.” (p. 47). He then continues that the “*stag, who inhabits the forests, and lives only upon wood, produces a species of trees, which is nothing more than the superabundant part of his food*” (pp. 49–50), and further that the “*horns, then, are but an excrescence, a part foreign to the body of the stag, and only esteemed as an animal substance because it grows from him; it is in reality a vegetable production, since it retains all the marks of that vegetable from which it derives its origin, and resembles the branch of a tree in the manner it grows, expands, hardens, dries, and separates; for it falls off spontaneously, after having acquired its full degree of solidity, like a ripe fruit from the branch*.” (p. 50; all quotes from the English translation by J.S. Barr, 1797 [214]).

On first sight, these sentences might be understood as indicating that Buffon considers antlers to be wooden structures. However, this would be a misinterpretation of his views. What Buffon actually means is that the abundant organic (plant) matter taken up as food retains some of its original properties when the stag subsequently uses this matter for antler production, and that, as a result, the antlers show traits also found in the trees from which this organic matter was originally derived. This means that the constituents of the stag’s food cause an “imprint” on the bony antlers that make them resemble trees. Expressed in Buffon’s words, it “*must be allowed, however, that organic matter, which produces these horns […] preserves even after it has passed through the body of the animal, characteristics of its former vegetable state*” ([214], p. 46) and “*therefore the organic molecules, which constitute the living substance of the horns of the stag, still retain the image of the vegetable, because they are arranged in the same manner as in vegetables. Here we see that matter has an influence over form*.” ([214], p. 48). Therefore, the statement by Goss ([2], p. 133) that Buffon “*believed that deer antlers were made of wood*” misrepresents Buffon’s views on this matter. Using different lines of argumentation, Buffon’s concept of the “imprint” of the stag’s food plants on his antlers were rejected by Cuvier [215,216] as well as by Blumenbach [217].

It has recently been argued that in his work Buffon implicitly distinguished between plants as a classificatory group and “vegetative reproduction” [218]. According to this interpretation, Buffon’s views on nutrition, development and reproduction are based on a “vegetal model” of processes common to animals and plants, and within this framework he considers some parts of animals (hairs, nails, horns and bones) as developing by way of vegetative reproduction [218]. Antlers can be added to this list as a special type of bony structure.

As already emphasized by Buffon [214], antler growth occurs in an appositional mode, and the oldest bone is therefore located at the antler base, while the youngest is present at the tips. This growth takes place in richly vascularized growth zones, also referred to as antler growth centers (AGCs) [86], that are located at the tips of the main beam and the antler tines [51,70,86,219,220,221,222,223,224,225]. The antler is produced at (almost) its final thickness right from the start, as there is only little bone apposition along the antler periphery from the perichondrium/periosteum during growth (see below). Growth plates and secondary ossification centers, which are typical features of long bone growth in mammals, are not present in antlers. There is also no initial formation of a small cartilaginous model of the antler, which again is in contrast to the situation in long bone development.

The nature of the “pre-osseous” tissue formed in the AGCs has long been a matter of debate [220,221,226]. Home ([95], p. 66) (correctly) stated that the growing antler tip is “*a vascular cartilaginous structure, covered with a velvet-like cutaneous covering*.” Lieberkühn [227] and von Korff [228] likewise described the initially formed tissue at the antler growth tips as cartilage, and concluded that the mode of antler ossification is endochondral. Gruber [70] used the term “epichondral growth” to describe the appositional activity of the chondrogenic growth zones at the antler tips, and also characterized the ossification mode in the antlers as endochondral. However, in a later paper, Gruber [229], possibly influenced by the work of Wislocki [96], uses the term “cartilage-like fiber-rich tissue” for the tissue formed at the antler growth tips. Other authors regarded the mode of bone formation in antlers of antlers as comparable to intramembranous ossification [96,230], while Wislocki et al. [226] later characterized the antler growth region as being composed of “chondroidal tissue.”

The seminal light and electron microscopic studies by Banks [219], Banks and Newbrey [220,221] and Newbrey and Banks [231] settled the controversy on the nature of the pre-osseous tissue formed during antler growth. These authors demonstrated that the tissue initially formed in the AGC is hyaline cartilage and concluded that antler bone formation in antlers constitutes a special type of mammalian endochondral ossification [221]. This view has since been corroborated by other groups, who added additional observations on the AGC [51,86,222,223,224,232,233]. Among the special features of the antler endochondral ossification system, the rich vascularization of the AGC is especially notable and constitutes a major difference to the avascular growth plates of mammalian long bones. The rich blood supply of the antler growth region provides the necessary oxygen and nutrients required for the exceptionally rapid cartilage formation during antlerogenesis [220,221,234].

**Figure 18:**
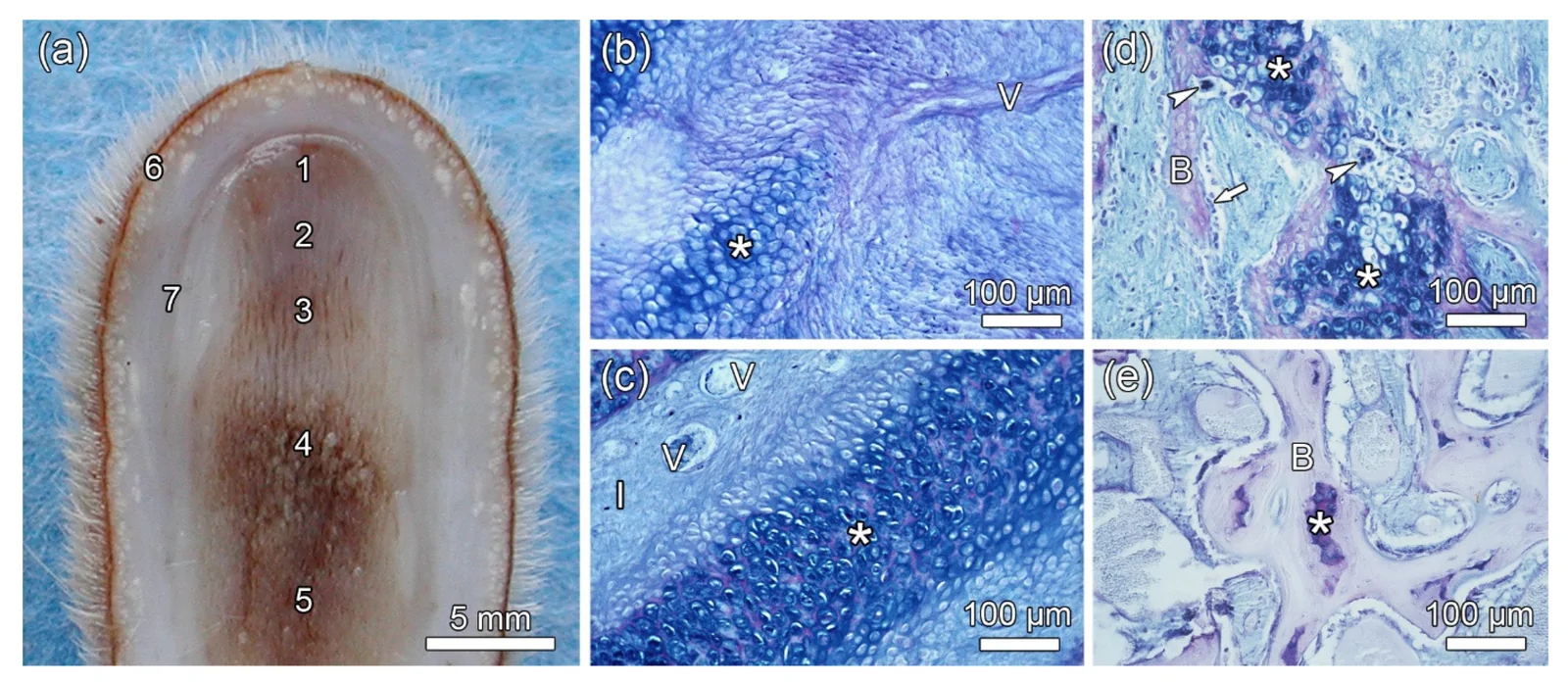
Macroscopic overview of longitudinally sectioned tip region of a growing first antler from a fallow deer (*Dama dama*) (**a**) and histological images (**b**–**e**) of different zones of the tip region of a growing fallow deer first antler (AB-PAS-stained longitudinal sections), modified after [222]. (**a**) The approximate location of the different zones in the growing antler tip is indicated by numbers, 1—proliferative zone, 2—zones of cartilage maturation and hypertrophy, 3—zone of cartilage calcification, 4—primary spongiosa, 5—secondary spongiosa. Also indicated are the antler velvet (6) and the periosteum/perichondrium (7). (**b**) Transition between prechondroblastic and chondroblastic region in the proliferative zone. Asterisk—early forming cartilaginous column, V—blood vessel. (**c**) Cartilaginous column (asterisk) from the zone of mature cartilage. While mature chondrocytes are present in the center of the column, young chondrocytes and chondroblasts are located more peripherally. I—richly vascularized intertrabecular tissue, V—blood vessels. (**d**) Primary spongiosa showing co-occurrence of resorption of cartilage (asterisks) by multinucleated chondroclasts (arrowheads) and deposition of bone tissue (B). Arrow—osteoblastic seam. (**e**) Remnants of calcified cartilage (asterisk) covered by newly deposited bone tissue (B).

The tip regions of growing antlers can in a distal-to-proximal sequence be divided into a proliferative zone, zones of cartilage maturation and hypertrophy, a zone of cartilage calcification, a primary spongiosa and a secondary spongiosa (Figure 18a) [220,221]. As will be detailed below, the various zones are not sharply demarcated from each other.

The proliferative zone can be subdivided from distal to proximal in a so-called reserve mesenchyme, the inner portion of which is densely populated by highly proliferative neural crest-derived mesenchymal stem cells, a prechondroblastic region and a chondroblastic region. Within the proliferative zone, the cells of the reserve mesenchyme undergo sequential differentiation into prechondroblasts and then chondroblasts (Figure 18b). The developing cartilage is organized into interconnected vertically oriented trabeculae (columns) that form a tubular framework (scaffold) around pore spaces occupied by richly vascularized intertrabecular connective tissue.

Proximal to the proliferative zone, the chondrocytes within the cartilaginous columns undergo maturation and then hypertrophy. The zones of maturation and hypertrophy are not clearly juxtaposed to each other, but within the cartilaginous columns there is both a differentiation gradient along the vertical axis and the central-to-peripheral axis. More distally, mature chondrocytes occupy mid-trabecular positions, while young chondrocytes and chondroblasts are located peripherally (Figure 18c). Further proximally, hypertrophic chondrocytes are first found in the center of the cartilaginous columns, while mature chondrocytes, young chondrocytes and chondroblasts are still present in gradually more peripheral positions. Like the hypertrophy of the chondrocytes, also the calcification of the cartilage matrix starts in mid-trabecular positions and from there spreads towards the periphery [220,222].

The primary spongiosa is mainly characterized by the co-occurrence of chondroclastic and osteoblastic activity (Figure 18d). Due to these activities, the calcified cartilage undergoes progressive reduction, and the initially formed longitudinally oriented, tubular cartilaginous framework is largely replaced by an osseous one with the same orientation. This osseous tubular framework consists partly of woven, partly of lamellar bone [3,228,235].

In the secondary spongiosa, the perivascular cavities lined by the trabecular framework, which on antler cross sections appears honeycomb-like, are gradually filled with elongated circular lamellar systems [3,103,108,235,236,237]. This infilling is most intense in peripheral regions, leading to the formation of a dense antler cortex with numerous primary osteons, while a markedly less-intense infilling occurs within the inner antler portion that forms the spongious antler core. Between cortex and spongiosa, a transition zone of intermediate density can often be discerned.

Due to their limited lifespan of only a few months, there is very little bone remodeling in antlers. Accordingly, antler bone contains only few secondary osteons that are mostly present in the basal (oldest) antler portions [96,103,108,235,236]. When the velvet antler period of fallow bucks was experimentally expanded by treatment with an antiandrogen, their antlers showed evidence of more intense remodeling activity in the form of a higher prevalence of secondary osteons and resorption spaces compared to control antlers with a normal duration of the velvet phase [237].

While the bulk of the cartilage formed in the AGCs is replaced by bone in a process of endochondral ossification, it has recently been suggested that a small subpopulation of antler hypertrophic chondrocytes transdifferentiate to osteoblasts via PHEX^+^ cells as intermediates [234]. The PHEX^+^ cells were located adjacent to hypertrophic chondrocytes and considered to likely represent chondrocytes that contribute to mineral deposition. Strong support for a chondrocyte-to-osteoblast transition during antler growth came from a xenogeneic transplantation experiment. Following AP transplantation into nude mice, large avascular cartilage nodules developed in the hosts. By day 30 after transplantation, osteoblasts started to appear in the center of these nodules without evidence of previous chondroclasia [234].

Chondrocyte-derived osteoblast formation has been confirmed during pre- and postnatal endochondral bone formation in somatic bones, mainly using in vivo transgenic mouse models, and gene expression profiling indicated the involvement of different signaling pathways (NOTCH, BMP, MAPK) in this process [238,239]. The suggested occurrence of a cartilage-to-bone phenotypic switch also during antler growth [234] would be in line with these results.

In addition to endochondral ossification, antler growth also involves some direct (intramembranous) bone deposition from the perichondrium/periosteum, which causes the formation of a peripheral bone layer [44,220,222]. This sleeve bone stabilizes the antler growth region, thus having a similar function as the sleeve bone formed by intramembranous ossification in the diaphyseal region of developing long bones. It has been shown that the small surface protuberances (pearls) of the outer cortex of roe deer antlers are also formed by this subperiosteal (intramembranous) ossification [108].

**Figure 19:**
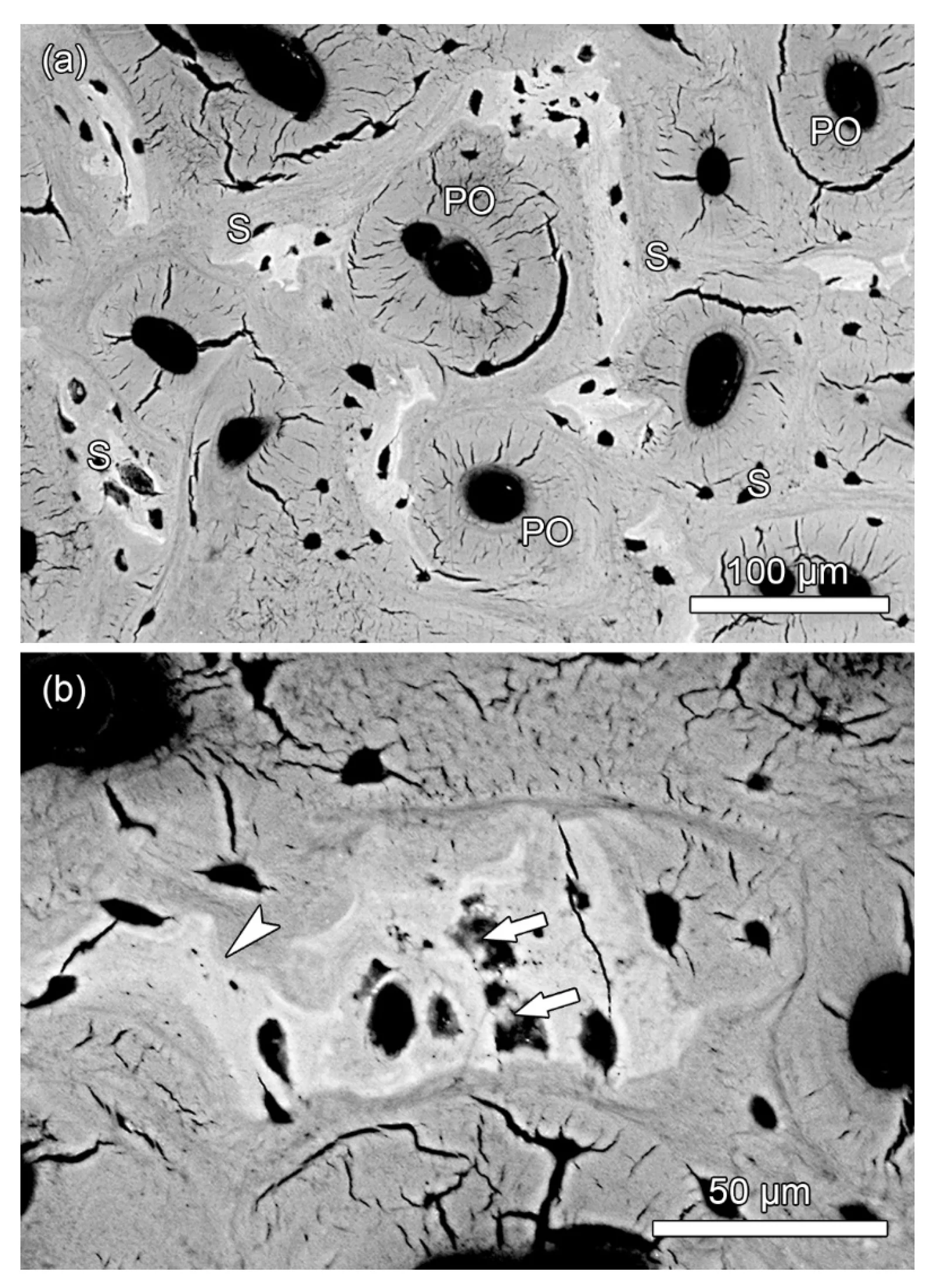
SEM-BSE images of polished block surfaces of transversely sectioned hard fallow deer antlers. (**a**) Section surface of distal antler portion showing remnants of (bright) calcified cartilage within the trabecular scaffold (S) and primary osteons (PO) that have filled the intertrabecular spaces. (**b**) Calcified cartilage remnant at higher magnification. The calcified cartilage appears brighter than the surrounding bone, denoting the former’s higher degree of mineralization. Also note the scalloped surface of the calcified cartilage (arrowhead) resulting from previous chondroclastic resorption and the presence of mineralized deposits (arrows) in some of the chondrocyte lacunae; for further explanation, see [241].

Some authors have stated that the calcified cartilage formed during early antler development is completely replaced by bone during later growth stages [217,220,235]. If this were the case, the situation in antlers would parallel the typical situation in other endochondrally forming bones in mammals, where the calcified cartilage is a transient tissue that eventually becomes completely replaced by bone tissue. However, several studies have demonstrated the presence of remnants of calcified cartilage in hard antlers of different deer species (Figure 18 and Figure 19) [108,240,241]. The persistence of calcified cartilage, like the scarcity of secondary osteons, can be attributed to the short-lived nature of antlers and the resulting virtual absence of bone remodeling. Remnants of calcified cartilage are more frequent in distal compared to proximal antler portions, suggesting that the period of chondroclasia prior to the onset of osteoblastic activity is shorter distally. Calcified cartilage is more highly mineralized than bone, and therefore calcified cartilage matrix appears brighter than calcified bone matrix on polished block surfaces viewed in the scanning electron microscope (SEM) using a backscattered electron (BSE) detector (Figure 19) [241].

## 8. Antlers and Tumors

The exceptional growth rate of antlers has prompted the question of whether they are at risk of undergoing carcinogenesis [132,139,242]. Steinwedel [242] even suggested that normal antler growth is comparable to that of malignant tumors, and that antler casting has evolved as a means to protect deer from the detrimental consequences of this growth. This view was already rejected by Gruber [70], who emphasized that antler growth is a well-coordinated developmental process that shows none of the growth abnormalities characterizing malignant tumors.

Other authors have characterized the abnormal outgrowths developing from the permanently velvet-covered antlers of castrated or hypogonadal male deer (Figure 8 and Figure 9) as a kind of tumor growth [129,132]. According to Olt [129], peruke antlers should be classified as a type of malignant tumor. In contrast, other authors concluded that the hyperplastic skin and bone outgrowths of castrate antlers could be classified as benign but not as malignant tumors, as they don’t exhibit typical signs of malignancy like invasive growth and metastasis (132,133,139]. Moreover, it has been reported that although castrate antlers show unrestricted growth, this growth is not permanent but shows a certain periodicity [121]. Also, castrate antlers of deer react to testosterone (or estrogen) administration like normal velvet antlers [121,122].

**Figure 20:**
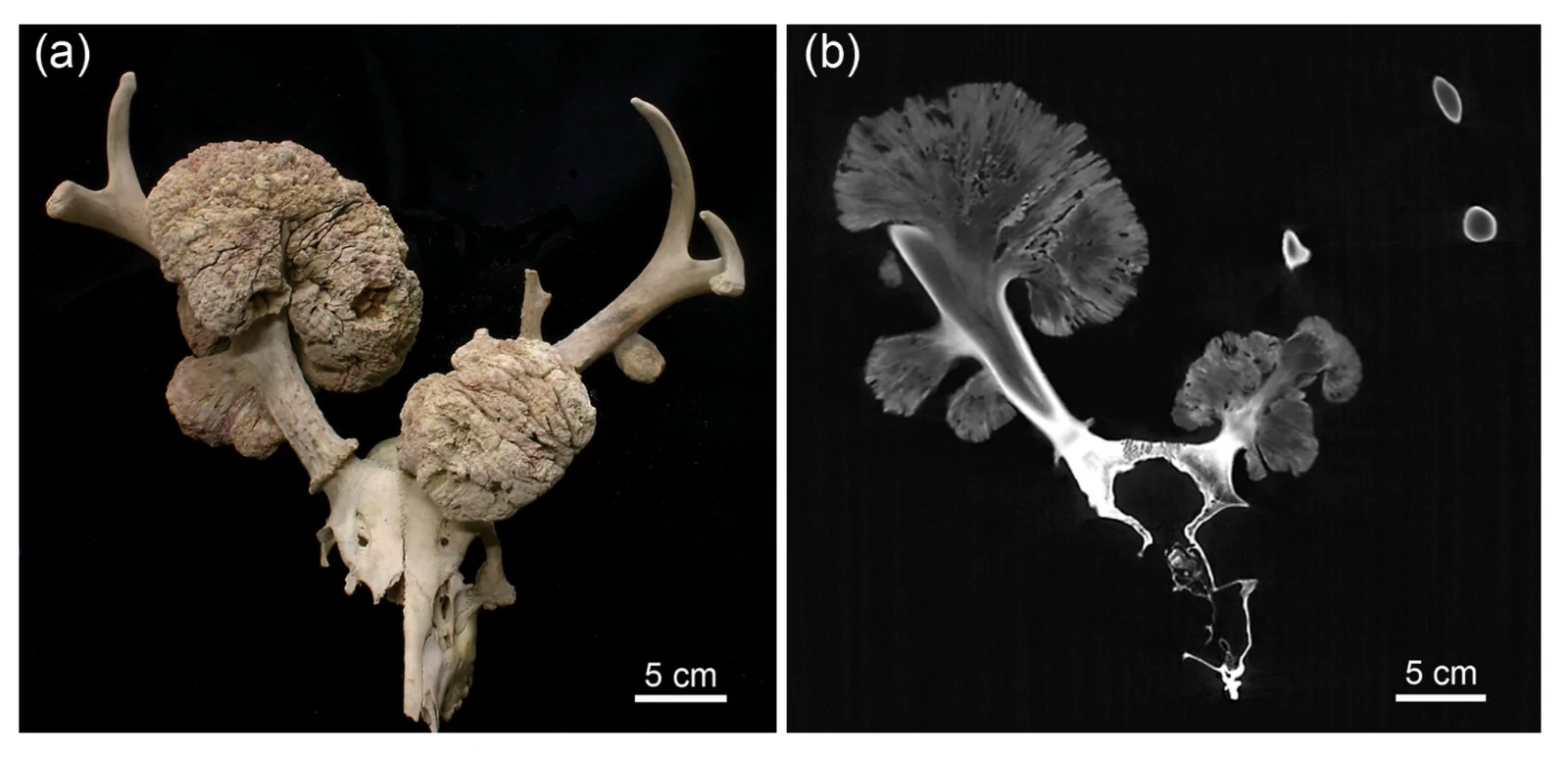
Multiple osteochondromas on the antlers of a white-tailed buck (*Odocoileus virginianus*). The cranium along with few postcranial bones was found by a hunter in the field in Georgia, USA [244]. (**a**) Anterior view of the cranium showing the large dimensions of the antler tumors and the otherwise normal morphology of the antlers. (**b**) CT-image (frontal section) of the cranium showing the lower density of the osteochondromas compared to the skull roof and antler cortex.

Only very few cases of formation of typical bone tumors also found in other mammal species have been reported in antlers [243,244]. These tumors were classified as osteochondromas, a type of benign tumor composed largely of osseous and cartilaginous tissues. Antler osteochondromas can reach a large size during the short antler growth period (Figure 20), which refutes the view by Goss [139] that the rarity of antler tumors in intact deer reflects the short lifespan of antlers, which is less than the time required by most tumors to become overt.

The various genes, proteins (growth factors, transcription factors, intracellular proteins, extracellular matrix proteins) and signaling pathways involved in the control of rapid antler growth have been intensely studied during more recent years [18,245,246,247,248,249,250], with a focus on the stimulation of chondrogenesis and the promotion of angiogenesis and nerve regeneration. A particular important finding by Wang et al. [18] was that three proto-oncogenes (*FOS*, *REL*, *FAM83A*) were under positive selection in antlers, and that five growth factor and receptor genes (*FGF19*, *FGF21*, *FGFbP3*, *PDGFD*, and *PDGFRL*), known to be important for cancer cell proliferation and survival, were highly and specifically expressed in growing antlers. The results of this study indicated that the gene expression profile of growing antlers is more similar to that of osteosarcomas than to that of normal bone tissue [18].

However, the study by Wang et al. [18] also showed that several tumor suppressor genes, including different cofactor and regulator genes of the p53 tumor suppressor, are under strong positive selection in deer [18]. Other studies have demonstrated a high level of apoptosis in the reserve mesenchyme of growing antlers [234,251] and high-level expression of apoptosis-related genes in mesenchymal cells from the AGC [234]. Furthermore, it was shown that MYC target genes of proliferating progenitor cells from the AGC differed considerably from those of osteosarcoma cells, suggesting that growing antlers use a non-tumorigenic transcription program [234].

These findings point to the action of a growth control mechanism in growing antlers that enables an enormous growth rate, while simultaneously preventing the malignant transformation of the rapidly proliferating cells in the AGC or eliminating transformed cells by apoptosis [10,18,234].

It was further shown that while most malignant tumors use glycolysis for ATP production, the cells from the antler’s reserve mesenchyme obtain their energy almost entirely by using the tricarboxylic acid (TCA) cycle and oxidative phosphorylation [10]. This is possible because the intense vascularization of the antler growth region provides the necessary oxygen for utilizing this metabolic pathway. It can thus be concluded that antlers effectively combine an unparalleled growth rate with a tight control of the growth process that largely eliminates the risk of carcinogenesis. In the rare cases of osteochondroma development in antlers, the cells forming these benign tumors can probably “profit” from the specific growth conditions present in antlers.

Starting from the observation of a tumor-suppressive environment in the antler growth region, several groups demonstrated an inhibitory effect of velvet antler preparations on the propagation of different types of cancer cells [9,10]. These findings hold the potential of providing new options for tumor treatment. Regarding the pathways involved in tumor control, a recent study showed that overexpression of an antler-derived micro-RNA (PC-3p-2869) reduced the proliferation, colony formation, and migration of human osteosarcoma and chondrosarcoma cells [11]. Another study demonstrated that engineered antler stem cell-derived exosomes could play a role in boosting anti-tumor immune responses, and thereby enhance tumor immunotherapy [12]. Future studies must show whether the findings from these studies can actually open up new avenues for cancer treatment in the clinical setting.

## 9. Conclusions

The principal aim of the present paper has been to give an overview of past and present ideas regarding certain basic aspects of antler biology, with a focus on antler initiation, growth, and regeneration. Other fields of antler research that have received increased attention in recent decades, including the evolutionary history of antlers [20,56], antler mineral composition [3,252] and biomechanics [3,253,254], the effects of nutrition and weather conditions on antlers [3,255,256] or the use of antlers in monitoring environmental pollution [257], are largely or completely outside the scope of this contribution.

In recent years, multi-omics studies have greatly advanced our understanding of the mechanisms involved in antler initiation, growth and regeneration, firmly establishing antler generation and regeneration as stem-cell based processes that are tightly controlled by gene regulatory networks, thereby enabling rapid growth while simultaneously preventing tumorigenesis. In addition, studies on the potential therapeutic use of antler stem cells or molecules released by these cells for improving wound healing, tissue repair and regeneration in humans add an important translational aspect to antler research [7].

Many questions regarding antler initiation, growth, and regeneration still remain unanswered, opening promising avenues for future research. Particularly interesting aspects to be addressed in future studies are (1) the identification and lineage tracing of future AP stem cells during embryonic development, (2) the interaction of systemic (endocrine, metabolic, and immunological) and local (tissue-specific, including exosomes) factors in casting wound healing and the control of antler growth, (3) the coordination of cell proliferation, tissue differentiation, vascularization and pattern formation in the AGCs, (4) further clarification of the mechanisms involved in the prevention of tumor formation during antler growth, (5) the overall control of antler morphogenesis, leading to the realization of species-specific antler shapes, (6) the role of chondrocyte-to-osteoblast transition in the process of antler bone formation, and (7) the similarities and differences between antler regrowth and other examples of epimorphic regeneration in vertebrates, such as fin regeneration in zebrafish, limb regeneration in amphibians, tail regeneration in lizards, and digit regeneration in mammals. Since the 1960s, the comparison of antler regrowth with other regenerative systems had been an important topic for Richard J. Goss [2,43,139,258], and it is in no small part thanks to him that antler regeneration is nowadays regularly covered in monographs on regenerative biology [259,260]. His attempts to establish antlers as a promising model system for studying organ regeneration in vertebrates were later continued by others [6,44,47,261,262,263,264,265]. In our view, a detailed comparison of the mechanisms governing antler regeneration and mammalian digit tip regeneration may in the future provide valuable insights for regenerative medicine.

As we have tried to demonstrate, many of the questions regarding the extraordinary attributes of antlers discussed today were already addressed in the past with the then available methods and under the prevailing conceptual frameworks of the time. We hope that by referring to these works we have convinced the reader that it is worthwhile to go back to the roots of antler research in order to better understand the origins of our current views on antlerogenesis and to give due credit to the accomplishments of our forerunners.

Learning from the past also means to be aware that our current views on antler development still have to stand the test of time, and that we must always be prepared to modify or even abandon our favorite hypotheses in the light of new evidence. Finally, we hope to have shown that antlers, and the history of antler research, are fascinating study subjects worthy of being pursued by researchers from different disciplines.

## Data Availability

All data are contained in the article.
